# Reciprocal EGFR signaling in the anchor cell ensures precise inter-organ connection during *Caenorhabditis elegans* vulval morphogenesis

**DOI:** 10.1242/dev.199900

**Published:** 2022-01-04

**Authors:** Silvan Spiri, Simon Berger, Louisa Mereu, Andrew DeMello, Alex Hajnal

**Affiliations:** 1 Department of Molecular Life Sciences, University of Zürich, Winterthurerstrasse 190, CH-8057 Zürich, Switzerland; 2 Molecular Life Science PhD Program, University and ETH Zürich, CH-8057 Zürich, Switzerland; 3 Institute for Chemical- and Bioengineering, Department of Chemistry and Applied Biosciences, ETH Zürich, 8093 Zürich, Switzerland

**Keywords:** *Caenorhabditis elegans*, Epidermal growth factor, Organogenesis, Microfluidics

## Abstract

During *Caenorhabditis elegans* vulval development, the uterine anchor cell (AC) first secretes an epidermal growth factor (EGF) to specify the vulval cell fates and then invades the underlying vulval epithelium. By doing so, the AC establishes direct contact with the invaginating primary vulF cells and attaches the developing uterus to the vulva. The signals involved and the exact sequence of events joining these two organs are not fully understood. Using a conditional *let-23* EGF receptor (EGFR) allele along with novel microfluidic short- and long-term imaging methods, we discovered a specific function of the EGFR in the AC during vulval lumen morphogenesis. Tissue-specific inactivation of *let-23* in the AC resulted in imprecise alignment of the AC with the primary vulval cells, delayed AC invasion and disorganized adherens junctions at the contact site forming between the AC and the dorsal vulF toroid. We propose that EGFR signaling, activated by a reciprocal EGF cue from the primary vulval cells, positions the AC at the vulval midline, guides it during invasion and assembles a cytoskeletal scaffold organizing the adherens junctions that connect the developing uterus to the dorsal vulF toroid. Thus, EGFR signaling in the AC ensures the precise alignment of the two developing organs.

## Abstract (precis)

**Summary:** A reciprocal EGF signal from the vulval precursor cells positions the invading anchor cell during *Caenorhabditis elegans* vulval development to link the vulva and uterus as they form.

## INTRODUCTION

Signaling by epidermal growth factor receptor (EGFR) tyrosine kinases controls a multitude of cellular processes during animal development (Segatto et al., 2011; Sisto et al., 2017; Yarden and Sliwkowski, 2001), with aberrant signaling resulting in various developmental defects and disease. Specifically, overactivation of EGFR signaling, caused by mutations in *EGFR* itself or in genes encoding downstream signal transducers, is known to contribute to the formation and progression of various cancer types in humans (Hsu and Hung, 2016; Rajaram et al., 2017; Sibilia et al., 2007). However, the role of deregulated EGFR signaling in cancer development is more complex than merely causing excess cell proliferation (Wee and Wang, 2017). Aberrant EGFR signaling in cancer cells promotes the formation of actin-rich protrusions, called invadopodia, and increases cell motility and invasion during metastatic growth, suggesting that secreted EGF ligands may act as chemoattractants (Goswami et al., 2005; Yamaguchi et al., 2005). By contrast, *Egfr* knockout (KO) mutations in mice perturb the morphogenesis of various organs, resulting in defective branching morphogenesis and alveolarization of the lungs and defects in heart morphogenesis (Chen et al., 2016; Iwamoto and Mekada, 2006).

In contrast to mammalian genomes, which encode four EGFR family members and multiple types of epithelial growth factors (EGF), the *Caenorhabditis elegans* genome encodes a single EGFR tyrosine kinase, LET-23, and one EGF-like protein, LIN-3 (Aroian et al., 1990; Hill and Sternberg, 1992). As in vertebrates, LET-23 EGFR signaling in *C. elegans* controls many different developmental processes (Sundaram, 2006). In particular, EGFR signaling during the development of the egg-laying system, composed of the vulva and uterus, has been studied in great detail (Gupta et al., 2012; Schindler and Sherwood, 2013). During vulval development, polarized LIN-3 EGF secretion from the AC towards the vulva precursor cells (VPCs) induces primary vulval cell fate by activating the EGFR/RAS/MAPK signaling pathway in P6.p, the nearest VPC, while simultaneously acting as an attractive cue inducing VPC migration towards the AC (Grimbert et al., 2016). The VPCs adjacent to P6.p, P5.p and P7.p receive less LIN-3 EGF signal and adopt a secondary vulval cell fate in response to a lateral Delta/Notch signal from P6.p, which inhibits primary fate adoption (Berset et al., 2001; Greenwald et al., 1983; Yoo et al., 2004). The distal VPCs (P3.p, P4.p and P8.p) only receive low doses of LIN-3 and Delta and adopt a tertiary non-vulval cell fate. After the VPC fates have been specified, P5.p, P6.p and P7.p undergo three rounds of cell division, generating 22 vulval cells.

Although not strictly required for vulval induction, the VPCs and their descendants secrete a LIN-3 isoform that depends on processing by the ROM-1 rhomboid protease to amplify the inductive AC signal via paracrine signaling (Dutt et al., 2004). In addition, LIN-3 expressed by the primary descendants of P6.p specifies the uterine-vulval1 (uv1) cells later during the L4 stage (Chang et al., 1999; Saffer et al., 2011).

During the L3 stage, after the vulval cell fates have been specified, the AC adopts an invasive phenotype and breaches the two basement membranes (BMs) separating the uterus from the developing vulva, establishing a direct connection between the two tissues (Sherwood and Sternberg, 2003). BM breaching requires the polarization of the actin cytoskeleton in the AC towards the VPCs aligned on the ventral midline. AC polarity is established by an UNC-6 Netrin signal from the primary VPCs, activating the Netrin receptor UNC-40 DCC in the AC and resulting in the recruitment of UNC-40 to the invasive membrane (Ziel et al., 2009; Naegeli et al., 2017). In addition, as-yet-unidentified diffusible guidance cues from the primary VPCs are required to attract the invading AC towards the vulval midline (Sherwood and Sternberg, 2003).

Following invasion, vulval morphogenesis begins with the apical constriction and invagination of the primary vulF cells. The other vulval cells migrate towards the vulval midline defined by the AC, extend circumferential protrusions and fuse with their contralateral partner cells, thereby forming a stack of seven syncytial rings, called the vulval toroids (Sharma-Kishore et al., 1999). Sequential contraction of the ventral toroids and expansion of the dorsal toroids give rise to a cylindrical lumen (Farooqui et al., 2012). Lastly, the vulF cells separate to expand the dorsal vulval lumen, and the AC occupies the space opened between the two vulF cells (Estes and Hanna-Rose, 2009). Consequently, the vulva is connected to the ventral uterus, and the AC fuses with the surrounding uterine cells to form the utse syncytium (Sapir et al., 2007). A robust connection between the vulva and uterus is indispensable for egg laying. However, the signals involved and the complete sequence of morphological changes that unite these two organs have not been fully resolved (Gupta et al., 2012).

Although the function of the LET-23 EGFR pathway in specifying vulval cell fates has been intensely investigated, possible roles of LET-23 signaling during vulval morphogenesis are not apparent, because they are likely masked by the earlier function of LET-23 during induction. The complete study of vulval development has additionally been hampered by the extended time period over which the process occurs and the often-subtle phenotypic differences caused by aberrant vulval morphogenesis. Therefore, we used the microfluidic long-term imaging approach introduced by Berger et al. (2021) to capture the entire process of vulval development in a continuous time series. Furthermore, to image large numbers of worms quickly and, therefore, detect subtle phenotypic differences across different larval stages, we developed an adaptation of this imaging approach, which allows for short-term, high-throughput imaging. Combining these two methods, we were able to reveal for the first time the complete sequence of cell rearrangements orchestrated by the AC. Moreover, using a conditional *let-23* allele, we show that LET-23 signaling in the AC is necessary to position the AC precisely and organize the formation of a new adherens junction between the AC and vulF toroid, connecting the developing vulva to the uterus.

## RESULTS

### LET-23 EGFR is expressed in the anchor cell during vulval morphogenesis

To study the tissue-specific functions of EGFR signaling, we previously constructed a conditional, GFP-tagged *let-23 egfr* allele [*FRT::let-23::FRT::gfp(zh131)*] by sequential CRISPR/Cas9-mediated insertion of two Flippase (FLP) recognition target sites (FRT) flanking the tyrosine kinase domain of *let-23* along with a *gfp* reporter near the C terminus (Konietzka et al., 2020). FLP-induced excision of the sequence flanked by the two FRT sites created a *let-23* loss-of-function (lf) allele and simultaneously led to the loss of the GFP signal. Given that an endogenously tagged *let-23::mKate2(re202)* allele has previously been reported to be a mild lf allele (Gauthier and Rocheleau, 2021), we scored vulval induction in *FRT::let-23::FRT::gfp(zh131)* animals to test whether insertion of the *gfp* tag or the FRT sites perturbed vulval development. We determined a vulval induction index (i.e. the mean number of induced VPCs per animal) of 3.0 for the *zh131* allele (*n*=44) as in the wild-type animals (*n*=44), indicating that the *zh131* allele has no adverse effect on vulval induction. The phenotypic discrepancy between the *zh131* allele used in this study and the *re202* allele reported by Gauthier and Rocheleau (2021) could result from the type of fluorescent reporter used in the LET-23 fusion proteins.

**Fig. 1.**
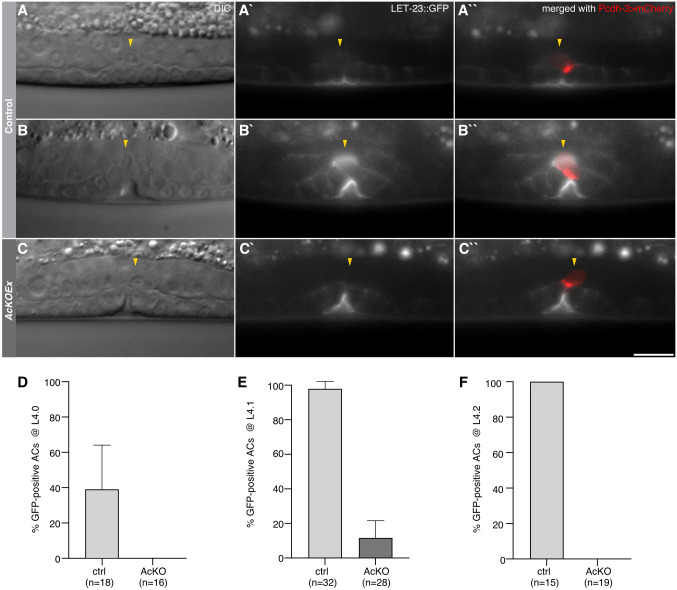
**Endogenous LET-23::GFP expression in the *Caenorhabditis elegans* AC.** (A-C″) *let-23::gfp(zh131)* reporter expression at the (A) L4.0 and (B) L4.1 substages of vulval development. (C) FLP recombinase-mediated deletion of *let-23::gfp(zh131)* using the *zhEx614[P_ACEL-pes-10_>2xNLS-FLP-D5]* extrachromosomal array, referred to as *let-23AcKOEx*. (A,B,C) DIC images of the mid-sagittal planes, (A′,B′,C′) LET-23::GFP signal in gray and (A″,B″,C″) LET-23::GFP signal overlaid with the *qyIs50[P_cdh-3_>mCherry::moeABD]* AC marker in red. Yellow arrowheads indicate the position of the AC. See Fig. S2 for additional examples and stages with the *zhIs146[P_ACEL-pes-10_>2xNLS-FLP-D5]* transgene. (D-F) Fraction of animals expressing LET-23::GFP in the AC without (ctrl) and with (AcKO) the *zhIs146[P_ACEL-pes-10_>2xNLS-FLP-D5]* transgene at the (D) L4.0, (E) L4.1 and (F) L4.2 substages. Error bars indicate the 95% CI and numbers in brackets the number of animals scored per condition. Scale bar: 10 µm.

While analyzing the expression pattern of the endogenous *let-23(zh131)* reporter, we not only observed the previously reported LET-23::GFP expression in the VPCs (Haag et al., 2014) and uv1 cells (Chang et al., 1999), but also detected LET-23::GFP expression in the AC, from the onset of vulval invagination at the L4.0 substage (Mok et al., 2015) until the AC fused with the ventral uterine (VU) cells (Fig. 1A-B″). The LET-23::GFP signal was localized diffusely in the AC near the contact site between the VPCs and uterus. To confirm the expression of LET-23::GFP in the AC, the *let-23(zh131)* allele was combined with a transgene expressing FLP driven by the AC-specific *lin-3* enhancer element and the minimal *pes-10* promoter (*zhEx614[P_ACEL-pes-10_>2xNLS-FLP-D5]*) (Hwang and Sternberg, 2004), hereafter referred to as *let-23AcKOEx*. In most *let-23AcKOEx* L4 larvae, the LET-23::GFP signal was absent in the AC without any obvious reduction in VPCs (Fig. 1C-C″), confirming expression in the AC in addition to the invaginating vulval cells during early vulval morphogenesis.

Initial analysis of vulval morphogenesis in *let-23AcKOEx* animals revealed a number of subtle and transient morphological defects, highlighting the importance of studying the dynamics of this process in real time as well as the necessity to image large numbers of worms rapidly.

### Short- and long-term microfluidic *C. elegans* imaging

Long-term *C. elegans* imaging was accomplished as described by Berger et al. (2021), continuously imaging worms from the early L3 stage up to adulthood, capturing the entire process of vulval development. Briefly, worms were housed in a parallel array of channels, continually fed and actively immobilized during image acquisition using a hydraulic valve. Channel dimensions were designed to confine animals only in height to maintain a stable orientation and animal identity, but otherwise allowing them to remain mostly free to grow and molt. Development on-chip, unlike on conventional agar pads, occurs reliably and with minimal negative effects on the observed process (Berger et al., 2021).

The acquisition of *z*-stacks of selected worms at single time points, here termed ‘short-term imaging’, is traditionally accomplished by immobilizing animals through compression on agar pads and by adding tranquilizing agents. Although simple in construction, agar pad-based imaging is typically laborious, with significant time spent on localizing and imaging randomly oriented animals. Previously, several microfluidic strategies for imaging of large worm populations have been introduced as an alternative. For example, Chung et al. introduced a sequential imaging method in which worms are automatically loaded into a single device (Chung et al., 2008; San-Miguel et al., 2016), whereas Mondal et al. (2016) proposed a parallel screening approach in which several thousand animals are trapped in a single large device and subsequently imaged.

**Fig. 2.**
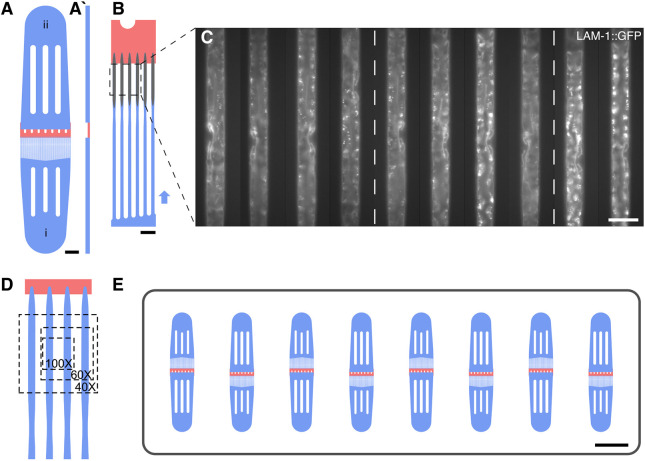
**Microfluidic short-term imaging device.** (A) Schematic overview of a single short-term imaging device. Worms are loaded at one end (i), with the excess fluid drained via the outlet (ii). (A′) Cross-sectional view (not to scale). The trap region (blue, bottom) is separated from the outlet region (blue, top) via a low-height region (red) effectively preventing worms from passing from one device compartment into another. (B) Magnified view of the device. Worms are manually loaded in the direction of the arrow, such that each of the 49 trap channels is filled with a single worm. Worm immobilization and orientation are achieved by carefully tuned device dimensions relative to the developmental stage studied, with the low-height region (red) stopping worms at the end of the trap channel. Once loaded, worms are kept in the imaging region by the tapered section at the tail end of each animal. (C) Representative images acquired on-chip showing a basement membrane marker *qyIs127[P_lam-1_>lam-1::gfp]* at the early- to mid-L4 stage. All worms are confined at the end of the trap channel and lined up in parallel, such that multiple worms may be imaged in one field of view (4 or 5 animals at 40× magnification, dashed box) and multiple fields of view may be imaged by moving along the device. (D) Magnified view of the parallel imaging scheme. The dashed overlays show the regions of interest achievable with different magnifications. (E) Schematic of a single microscope slide with eight individual devices capable of housing 392 animals simultaneously. Scale bars: 50 µm in C; 100 µm in B; 1000 µm in A; 5000 µm in E.

Both these methods allow imaging of large worm populations but require dedicated microscope setups and peripherals (e.g. pumps, solenoid valves or dedicated control electronics), rendering adaptation of either method as a routine imaging tool difficult. Here, we developed a parallel short-term imaging method, which is quick and easy to use for routine imaging, compatible with any microscope setup, without the need for additional equipment and, therefore, suitable as an effective replacement for agar-pad immobilization in routine imaging. Compared with the original long-term imaging device (Berger et al., 2021), the short-term device layout is simplified with an array of 49 parallel trap channels between a single inlet and outlet (Fig. 2A). Immobilization in these devices is achieved passively through a combination of channel height relative to width (ratio H/W 0.7-0.8), with channels closely following the size and shape of a trapped worm at the desired developmental stage, leaving little-to-no room for animal motion or growth. Although no active immobilization is used, immobilization can be further improved through addition of tetramisole or other chemical tranquilizing agents. Device dimensions for late L3 stage and mid-late L4 stage animals were chosen as 500×20×25 µm (type L3s; Supplementary file 1) and 600×22×30 µm (type L4s; Supplementary file 2) (L×H×W), respectively. The channel cross-section is kept constant, only tapering at the head and tail region, stabilizing these parts of the worm body and preventing worms from leaving the channel once loaded. All worms are trapped at the end of each channel, in a well-defined imaging region, by a drastic change in height (Fig. 2A-B, in red; Supplementary file 3). Given the regular arrangement of the trap array and the straight orientation of the worm, images of multiple animals can be acquired in parallel at high throughput with little time spent on localizing animals before image acquisition. Channel spacing was chosen such that, in the field of view (FOV) of a sCMOS camera with an 18.7 mm diagonal chip area, at 100× magnification two, at 60× three and at 40× five animals can be imaged simultaneously, with all 49 animals imaged in as few as ten FOVs (Fig. 2D). Acquisition speed may further be increased with eight identical devices fabricated on a single microscope slide, allowing imaging of up to 392 animals in quick succession, limited only by the acquisition speed of the microscope (Fig. 2E).

Device operation is even simpler than in the long-term imaging variant, such that a large number of worms can be loaded in only a few minutes. Synchronized worms are washed off the Nematode Growth Medium (NGM) plates and then loaded into the channels using a syringe connected to the inlet of a specific device through a short piece of tubing (see supplementary Materials and Methods for a detailed step-by-step protocol). Worms passively fill the trap channels one by one, because device dimensions are chosen that closely resemble those of the desired worm stage, resulting in a loading efficiency of ∼95% with all loaded animals trapped in lateral orientation. Once loaded, the tubing is removed and the device can easily be mounted on any microscope (upright and inverse). Importantly, animals remain viable for several hours on-chip without adverse effects of the device on worm health or morphology. In fact, short-term imaging devices may even be used for acquisition of 1- to 2-h-long time-lapse sequences, with trapped worms feeding on bacteria loaded along with the animals.

All devices are fabricated from highly transparent PDMS and mounted on cover glass, such that images may be acquired using high magnification and high numerical aperture (NA) objectives, and in combination with most commonly used imaging modalities (widefield and confocal microscopy) without any noticeable drop in image quality, compared with animals imaged on agar pads (Fig. 2C). Consequently, all images shown in this study, except for those in Fig. 1 and Fig. S1, were acquired using either the short- or long-term microfluidic imaging devices.

### LET-23 expression in the AC ensures precise alignment of the AC with the invaginating primary vulval cells

LIN-3 EGF is secreted by the primary vulval cells as a relay signal to ensure robust vulval induction during the L3 stage (Dutt et al., 2004) and to specify the uterine uv1 cell fate later during the L4 stage (Chang et al., 1999). Therefore, we investigated whether LET-23 EGFR might also function in the AC receiving a reciprocal LIN-3 signal from the primary VPCs.

For the following experiments, a stable *let-23AcKO* mutant strain was generated by combining the conditional *let-23(zh131)* allele with a single-copy integrated FLP transgene *zhIs146[P_ACEL-pes-10_>2xNLS-FLP-D5]*, hereafter referred to as *let-23AcKO* (Frøkjær-Jensen et al., 2008). To probe the activity and specificity of FLP expression, the *zhIs146* transgene was crossed with the heat shock-inducible FLP activity reporter *bqSi294[P_hsp16.41_>FRT::mCherry::his-58::FRT::GFP::his-58]* (Muñoz-Jiménez et al., 2017), which converts mCherry fluorescence into a nuclear GFP signal upon heat shock in cells with an active FLP. The *zhIs146* transgene induced conversion of the *bqSi294* reporter in the AC with 100% (*n*=20) efficiency (Fig. S1A,C). In 80% (*n*=20) of the animals, one or two of the adjacent VU descendants were also converted, probably because of the weak activity of the *ACEL-pes-10* enhancer/promoter during AC/VU specification during the early L2 stage (Fig. S1A,D). By contrast, FLP activity of the *zhIs146* transgene was never observed in the VPCs (Fig. S1A,B).

Faint LET-23::GFP expression in the AC was observed beginning at the L4.0 substage (Mok et al., 2015) in 39% (*n*=18) of control animals lacking the *zhIs146* transgene, compared with 0% (*n*=16) of *let-23AcKO* mutants (Fig. 1A,D; Fig. S2A-B′). During the following L4.1 substage, 98% (*n*=32) of the control animals compared with 12% (*n*=28) of *let-23AcKO* mutants showed AC expression of LET-23::GFP (Fig. 1B,E; Fig. S2C-D′), and in L4.2 larvae, the last substage before the AC fuses with the utse, AC expression was observed in all controls (*n*=15) and in none of the *let-23AcKO* mutants (*n*=19) (Fig. 1F; Fig. S2E-F′). No LET-23::GFP expression in other uterine cells besides the AC could be detected before the L4.3 substage, when LET-23::GFP expression was first observed in the uv1 descendants of the VU cells in 76% (*n*=13) of control animals (Fig. S2I). uv1 expression of LET-23::GFP was absent in 80% (*n*=42) of *let-23AcKO* animals by the L4.4 substage, probably because of earlier activity of *zhIs146* in the VU cells, as mentioned above (Fig. S1A,D and Fig. S2G-H′,J). Given that the AC is the only cell in the ventral uterus that expresses LET-23 during vulval invagination and toroid formation (before L4.3), the phenotypes induced by the ACEL-FLP transgene described in the following are most likely the result of the loss of LET-23 function in the AC.

**Fig. 3.**
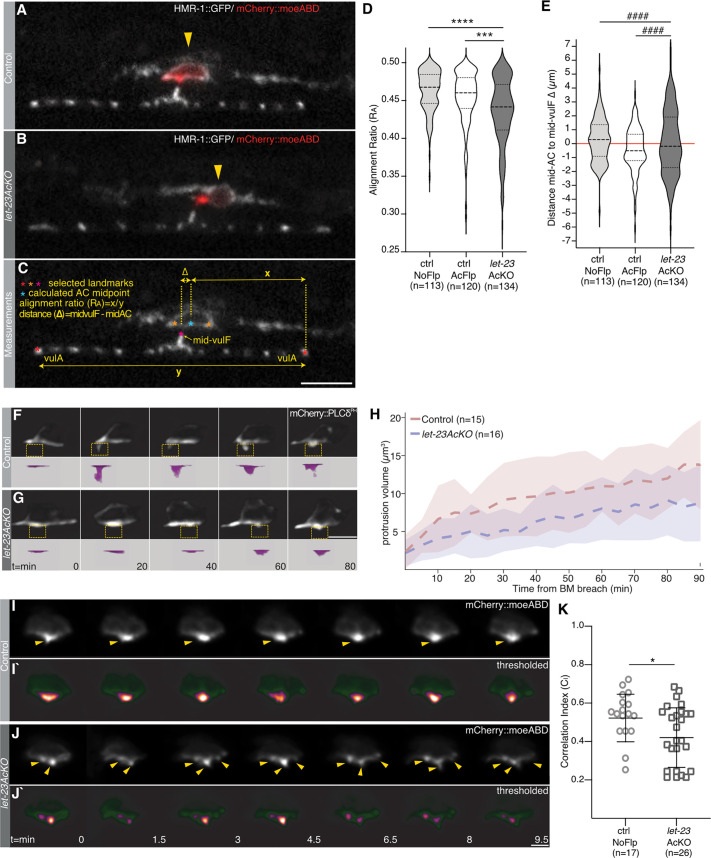
**Imprecise AC alignment and disorganized actin protrusions after AC inactivation of LET-23.** (A,B) Mid-sagittal planes of the HMR-1::GFP (*cp21*) adherens junction marker in white overlaid with the *qyIs50[P_cdh-3_>mCherry::moeABD]* AC marker in red in (A) a *let-23(zh131)* control animal lacking the *zhIs146[P_ACEL-pes-10_>2xNLS-FLP-D5]* transgene and (B) a *let-23AcKO* animal at the L4.0 stage. (C) Illustration of the landmarks (red, orange and magenta asterisks) selected to calculate the AC alignment index R_A_ and the absolute mid-AC to mid-vulF distance Δ. The blue asterisk indicates the AC center (mid-AC) calculated as the mid-point between the two HMR-1::GFP punctae defining the AC edges (orange asterisks). (D,E) Violin plots of (D) the AC alignment index R_A_ in *zh131*-negative control (ctrl NoFlp), *zhIs146*-negative control (ctrl AcFlp) and *let-23AcKO(zh131; zhIs146)* animals at the L4.0 stage and (E) the absolute distance mid-AC to mid-vulF in *zh131*-negative control, *zhIs146*-negative control and *let-23AcKO* animals. (F,G) Time series of the *qyIs24[P_cdh-3_>mCherry::PLCδ^PH^]* AC protrusion marker in (F) *zh131* control and (G) *let-23AcKO* larvae after BM breaching acquired at 5 min intervals. Every fourth frame is shown. See Movies 1 and 2 for all time points. Yellow-dashed boxes outline the invasive protrusions, shown as 3D views in magenta in the bottom row (magnified ∼2.5-fold). (H) Increase in AC protrusion volume over time for *zh131* control (red) and *let-23AcKO* (blue) animals. Dashed lines indicate the mean protrusion volume, with faded colors indicating ±1 s.d. from mean. (I,J) Time series of the *qyIs50[P_cdh-3_>mCherry::moeABD]* actin reporter in the AC in (I) a *zh131* control and (J) a *let-23AcKO* larva at the L4.0 stage acquired at 30 s intervals. Every third frame is shown. See Movies 3 and 4 for all time points. Yellow arrowheads in I indicate the single AC protrusion formed at the midline in a control animal and, in J, the multiple AC protrusions formed in *let-23AcKO* animals. (I′,J′) Heat-maps of the binarized AC images from I,J used to calculate the frame-to-frame correlation indices C_I_. (K) Mean correlation indices C_I_ measured in the AC of *zh131* control and *let-23AcKO* L4.0 animals. Bars indicate the mean±s.d. Dashed lines in the violin plots in D,E indicate the median values and the dotted lines the upper and lower quartiles. Statistical significance was calculated in D with a Student's *t*-test for independent samples of unequal variance and in K with a nonparametric, unpaired Mann–Whitney *U* test because the data were not normally distributed (***P<0.001, ****P<0.0001). ^####^ in E indicates the result of an *F*-test for variance, indicating unequal variance with *P*<0.0001. Numbers in brackets below each graph refer to the number of animals scored per genotype. Scale bars: 5 µm in G,J′; 10 µm in C.

We analyzed vulval morphogenesis in *let-23AcKO* mutants using the endogenous cadherin reporter *hmr-1::gfp(cp21)* to visualize the adherens junctions together with the actin marker *qyIs50[P_cdh-3_>mCherry::moeABD]* labeling the AC (Marston et al., 2016; Ziel et al., 2009). This analysis revealed a displacement of the AC from the vulval midline in *let-23AcKO* mutants at the L4.0 substage, the first time point in which AC expression of LET-23::GFP was detected (Fig. 3A,B). We quantified AC positioning in each animal by calculating an alignment ratio (R_A_), defined as the relative position of the AC mid-point to the invaginating vulval cells (with R_A_=0.5 indicating perfect centering), as well as by measuring the absolute distance (Δ) between the AC and vulF mid-points (Fig. 3C; see Materials and Methods). In control *let-23(zh131)* animals lacking the *zhIs146* FLP transgene (ctrl NoFlp), the mean R_A_ was 0.462±0.028 (*n*=113), and in *zhIs146* animals lacking the *let-23(zh131)* allele (ctrl AcFlp), the mean R_A_ was 0.454±0.037 (*n*=120), indicating close alignment of the AC with the invaginating vulval cells. By contrast, AC alignment in *let-23AcKO* mutants was less precise, with a mean R_A_ value of 0.435±0.045 (*n*=134) and a higher variability of R_A_ (Fig. 3D). In addition, the absolute distance Δ showed an increased variance in *let-23AcKO* mutants compared with the two controls (Fig. 3E: s.d. control NoFlp: ±1.584 µm; s.d. control AcFlp: ±1.619 µm; s.d. *let-23AcKO*: ±2.380 µm).

To test whether the observed AC positioning defect was caused by a depolarization of the AC upon inactivation of LET-23, we measured the dorsoventral polarity index (I_DV_) using the *qyIs50[P_cdh-3_>mCherry::moeABD]* reporter as described (Mereu et al., 2020; see Materials and Methods). This analysis revealed a slightly increased, rather than decreased, AC polarity index in *let-23AcKO* mutants, suggesting that LET-23 signaling is not necessary to polarize the AC (Fig. S3A).

Next, we performed confocal time-lapse imaging to analyze the dynamics of the invasive AC protrusion that forms shortly after BM breaching in mid-L3 larvae (Naegeli et al., 2017). We imaged *let-23AcKO* mutants and control animals carrying the *qyIs24[P_cdh-3_>mCherry::PLCδ^PH^]* reporter labeling the actin-rich AC protrusions combined with the *qyIs10[P_lam-1_>lam-1::GFP]* BM marker at 5 min intervals in animals trapped in long-term microfluidic imaging devices (type L2-A). This analysis revealed that the invasive AC protrusions were overall smaller and less focused in *let-23AcKO* mutants compared with control animals lacking the *zhIs146* FLP transgene (Fig. 3F,G; Movies 1 and 2). We quantified the total volume of the main invasive protrusion formed during the first 90 min after the AC breached the BM in control (*n*=15) and *let-23AcKO* mutants (*n*=16), as described by Kelley et al. (2017) and Naegeli et al. (2017). The volume and growth rate of the AC protrusion were both reduced in *let-23AcKO* mutants compared with control animals (Fig. 3H; Fig. S3B,C).

To capture the dynamics of the actin cytoskeleton at higher temporal resolution, we performed rapid time-lapse imaging experiments in L4.0 animals after the BMs had been breached. We imaged the *qyIs50[P_cdh-3_>mCherry::moeABD]* actin reporter at 30 s intervals for a total of 10 min in animals trapped in the short-term devices. In most control animals, the actin reporter signal was concentrated in a single dot on the ventral AC surface, directed towards the mid-point of the invaginating vulF cells. In the AC of *let-23AcKO* mutants, we observed several actin foci in multiple protrusions (Fig. 3I-J′; Movies 3 and 4). The dynamic changes in the AC were quantified through correlation analysis of consecutive time points, effectively assessing the degree by which AC morphology changes over time (see Materials and Methods). This analysis indicated a more dynamic rearrangement of the AC (i.e. a lower correlation index C_I_) in *let-23AcKO* mutants (Fig. 3K) compared with controls.

In summary, our data indicate that LET-23 expression in the AC is required for the precise alignment of the AC with the invaginating vulval cells. LET-23 signaling might be necessary to organize the actin cytoskeleton in the AC, forming the invasive protrusion directed towards the vulval midline.

### LET-23 signaling in the AC promotes basement membrane breaching

Over the course of vulval organogenesis, the AC breaches two BMs to establish a physical connection between the developing vulva and uterus (Sherwood and Sternberg, 2003). During this process, the UNC-40 DCC receptor in the AC is activated by an UNC-6 Netrin signal from the primary VPCs to polarize and guide the AC towards the underlying vulval cells (Ziel et al., 2009; Naegeli et al., 2017).

In wild-type animals, the BMs are completely breached before vulval invagination begins at the L4.0 substage, because AC invasion normally occurs during mid-L3 (the Pn.pxx stage). In *unc-6(lf)* mutants, the BMs are only breached in ∼30% of the animals at the L4.0 substage, whereas, by the L4.4 substage, BM breaching has occurred in ∼80% of the animals, indicating delayed AC invasion (Ziel et al., 2009). Therefore, it has been proposed that UNC-6 Netrin acts in parallel with one or several additional diffusible guidance cues secreted by the primary VPCs (Sherwood and Sternberg, 2003).

**Fig. 4.**
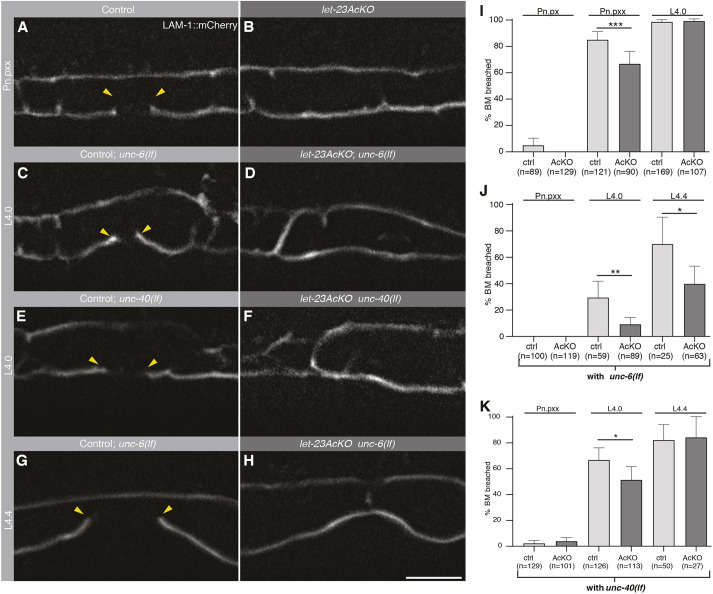
**BM breaching defects after AC-specific LET-23 inactivation.** (A,B) Mid-sagittal planes showing expression of the *qyIs127[P_lam-1_>lam-1::mCherry]* BM marker in (A) a *let-23(zh131)* control animal lacking the *zhIs146[P_ACEL-pes-10_>2xNLS-FLP-D5]* transgene and (B) a *let-23AcKO* animal at the Pn.pxx stage of the L3 stage. (C-H) LAM-1::mCherry expression in (C) *let-23(zh131); unc-6(ev400lf)* control, (D) *let-23AcKO; unc-6(ev400lf)*, (E) *let-23(zh131); unc-40(e271lf)* control and (F) *let-23AcKO; unc-40(e271lf)* animals at the L4.0 substage (beginning of invagination) and (G) in *let-23(zh131); unc-6(ev400lf)* control and (H) *let-23AcKO; unc6(ev400lf)* animals at the L4.4 substage (completed invagination). The yellow arrowheads in A,C,E,G indicate the extent of the BM breach. (I,J,K) Frequency of BM breaching at the indicated stages in (I) *zh131* control (ctrl) and *let-23AcKO* animals, (J) *let-23(zh131); unc-6(ev400lf)* control and *let-23AcKO; unc-6(ev400lf)* animals and (K) *let-23(zh131); unc-40(e271lf)* control and *let-23AcKO; unc-40(e271lf)* animals. Error bars indicate the 95% CI and numbers in brackets the number of animals scored per condition. Statistical significance was calculated using a nonparametric, unpaired Mann–Whitney *U* test (*P<0.05, **P<0.01, ***P<0.001). Scale bar: 10 µm.

Given that the loss of LET-23 function affects AC alignment, invasive protrusion formation and actin dynamics, we tested whether LET-23 signaling in the AC may act in parallel with UNC-6 to ensure efficient BM breaching. For this purpose, we observed *let-23AcKO* mutants carrying an *mCherry*-tagged *lam-1 laminin* reporter (*qyIs127[P_lam-1_>lam-1::mCherry]*) (Ihara et al., 2011). *let-23AcKO* single mutants exhibited a slight but significant delay in BM breaching at the Pn.pxx stage, because only 64% of *let-23AcKO* animals showed a gap in their BMs, compared with 83% of control animals (Fig. 4A,B,I). However, by the L4.0 substage, the BMs were breached in all *let-23AcKO* mutants (Fig. 4I). This suggested that, although not being essential for invasion, LET-23 signaling in the AC might cooperate with other guidance cues to promote BM breaching. Thus, we analyzed AC invasion in *let-23AcKO; unc-6(ev400lf)* double mutants. Only 11% of *let-23AcKO; unc-6(ev400lf)* double mutants at the L4.0 substage exhibited BM breaching, compared with 29% of *unc-6(ev400lf)* single mutants (Fig. 4C,D,J). By the L4.4 substage only 40% of the *let-23AcKO; unc-6(ev400lf)* double mutants showed BM breaching, compared with 68% of *unc-6(ev400lf)* single mutants (Fig. 4G,H,J). In addition, in *let-23AcKO; unc-40(e271lf)* double mutants, we observed a reduced frequency of breached BMs compared with *unc-40(e271lf)* single mutants at the L4.0 substage (Fig. 4E,F,K). However, by the L4.4 substage, there was no significant difference in BM breaching between *unc-40(e271lf)* single and *let-23AcKO; unc-40(e271lf)* double mutants.

We conclude that LET-23 signaling in the AC transduces a guidance cue that cooperates with the UNC-6 Netrin signal secreted from the VPCs to achieve efficient BM breaching. Given that LIN-3 EGF is only expressed by the primary vulF cells at this stage (Chang et al., 1999; Saffer et al., 2011), it seems likely that LET-23 in the AC receives the LIN-3 signal from the invaginating vulval cells.

### Inactivation of LET-23 in the AC causes disorganized vulval-uterine junctions

AC invasion and alignment are crucial to initiate dorsal lumen formation during the subsequent stages of vulval morphogenesis (Estes and Hanna-Rose, 2009). After invasion, the AC remains positioned at the vulval midline in a pocket surrounded by the invaginating vulF cells. If the AC fails to invade into the vulval tissue or if it is displaced, an abnormal dorsal vulval lumen will form (Estes and Hanna-Rose, 2009). Therefore, we investigated the consequences of the early AC positioning defect observed in *let-23AcKO* mutants at the L4.0 substage on vulval lumen morphogenesis.

To obtain detailed insights into the dynamics of vulval morphogenesis, we used long-term imaging devices (type L2-A, Berger et al., 2021), which allowed seamless imaging of the same animal from the early L3 stage until adulthood. Vulval morphogenesis was observed in control and *let-23AcKOEx* animals carrying the *hmr-1::gfp(cp21)* and *qyIs50[P_cdh-3_>mCherry::moeABD]* reporters, outlining the adherens junctions and AC, respectively (Movies 5 and 6).

**Fig. 5.**
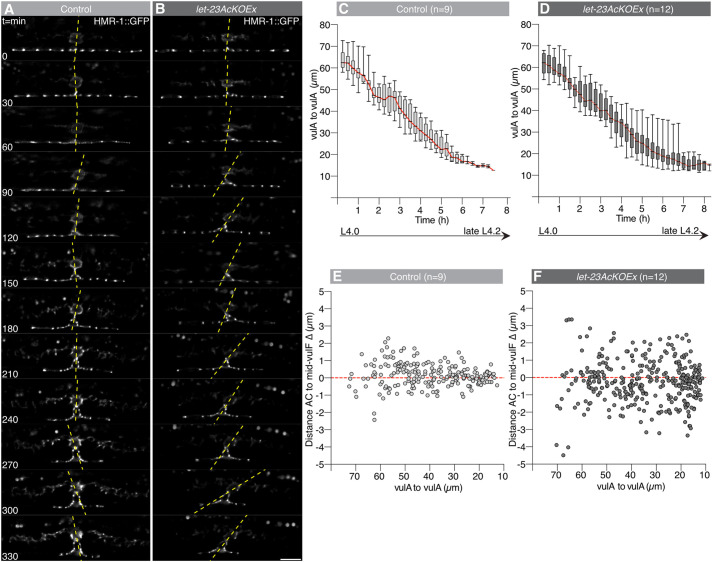
**Variable AC position during vulval invagination after LET-23 inactivation.** (A,B) Time series showing the mid-sagittal plane for the HMR-1::GFP (*cp21*) adherens junction marker between the L4.0 and late L4.2 substages in (A) a *let-23(zh131)* control and (B) a *let-23AcKOEx* animal. Yellow-dashed lines are drawn through the mid-vulF point and the calculated AC mid-point. Images are shown at 30 min intervals. (C,D) Box and whisker plots showing the declining distance between the outer vulA junctions over time, used as a measure of vulval cell migration to the midline. The red lines connect the medians throughout the time points. Box plots in C and D show the median (black lines) with upper and lower quartiles (boxes), whiskers indicate the most extreme points of the distribution. (E,F) Dot plots showing the absolute mid-AC-to-mid-vulF distances Δ relative to the vulA-to-vulA distance. The numbers in brackets above each graph indicate the number of animals scored per genotype. See also Fig. S3 for the individual measurements over time for each animal. Scale bar: 10 µm.

These experiments showed that the AC in control animals remained fixed after it had made initial contact with the invaginating vulF cells (Fig. 5A). By contrast, the AC position in *let-23AcKOEx* mutants was highly variable, causing the connected toroids to rock back and forth along the anterior-posterior (AP) axis (Fig. 5B). In the following, we assessed the robustness of vulval morphogenesis by measuring vulA migration and found that the distance between the outer vulA junctions decreased at a similar rate in both strains, indicating that the migration of the vulval cells towards the midline was not affected by loss of LET-23 function in the AC (Fig. 5C,D). The distance between the AC and vulF mid-points (Δ), plotted against the vulA distance as an indicator of developmental time, remained large and variable in *let-23AcKOEx* mutants (*n*=12), whereas it continually decreased in control animals (*n*=9) prior to AC fusion (Fig. 5E,F; Fig. S4A,B).

**Fig. 6.**
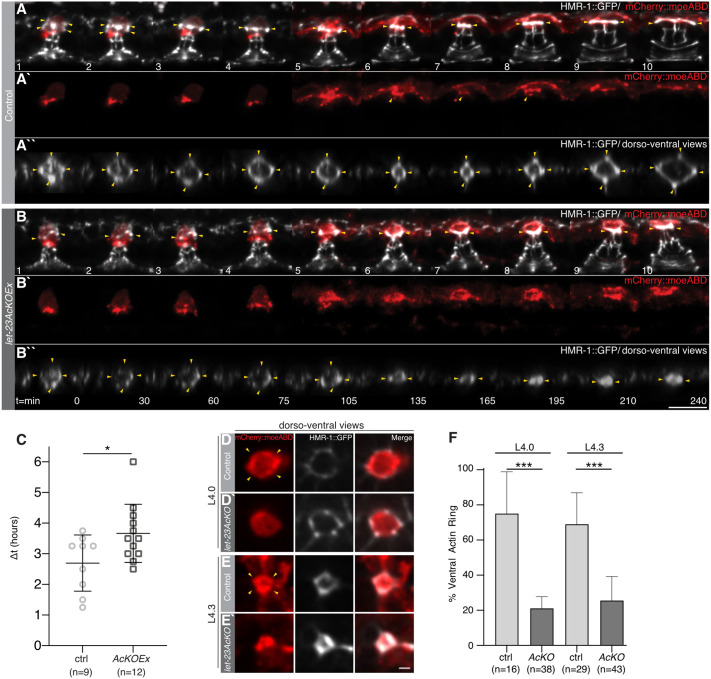
**Vulval toroid morphogenesis and actin ring assembly after AC-specific LET-23 inactivation.** (A-B″) Time series of the HMR-1::GFP (*cp21*) adherens junction marker in white overlaid with the *qyIs50[P_cdh-3_>mCherry::moeABD]* AC marker in red in a *let-23(zh131)* control (ctrl) (A) and a *let-23AcKO* animal (B) between the L4.1 and L4.4 substages (end of dorsal lumen expansion in ctrl). A1-10 and B1-10 show maximum intensity *z*-projections at the indicated time points. A′1-10 and B′1-10 show the P_cdh-3_>mCherry::moeABD actin marker in the AC. Yellow arrowheads in A1-10 and B1-10 point to the developing adherens junctions in the AC and, in A′6-8, to the actin cytoskeleton near the adherens junctions. A″1-10 and B″1-10 show dorsoventral views (cropped *x*,*z*-projections) of the AC-vulF interphase, with yellow arrowheads indicating the developing adherens junctions. (C) Time from AC fusion to the start of dorsal lumen expansion, between the L4.2 and L4.3 substages. Δt was defined as the time period between the last time point before the beginning of AC fusion (A4), indicated by the diffusion of the mCherry::moeABD signal into adjacent uterine cells, and the first time point at which an expansion of the dorsal adherens junction ring was observed (A″8). See Movies 5 and 6 for all time points and Fig. S5 for additional examples. (D-E′) Confocal images of the P_cdh-3_>mCherry::moeABD actin marker (red), the HMR-1::GFP (*cp21*) adherens junction marker (white) and merged images at the AC-vulF interphase in ventrally oriented (D) *zh131* control and (D′) *let-23AcKO* animals at the L4.0 and (E,E′) at the L4.3 substage. See Fig. S6 for additional examples. (F) Fraction of *zh131* control and *let-23AcKO* animals at the L4.0 and L4.3 substage containing a defined actin (semi)circle at the AC-vulF interphase. Horizontal black lines in C indicate the mean; error bars in C,F indicate the 95% CI. Statistical significance was calculated in C with a Student's *t*-test for independent samples of unequal variance and in F with a nonparametric, unpaired Mann–Whitney *U* test (*P<0.05, ***P<0.001). Numbers in brackets below each graph indicate the number of animals scored per genotype. Scale bars: 2 µm in E′; 10 µm in B″.

After generating 3D reconstructions of the time-lapse recordings, we noticed that bright HMR-1::GFP punctae appeared in the AC body dorsal to the actin-rich AC protrusion, which first contacts the vulF cells, indicating that recently developed adherens junctions were assembling in the AC (Fig. 6A-A″, yellow arrowheads in frames 1 and 2; Fig. S5A). Subsequently, a ring-shaped structure emerged from these adherens junctions (Fig. 6A-A″, yellow arrowheads in frames 3 and 4), which contracted as it joined with the adherens junctions on the dorsal surface of the vulF toroid (Fig. 6A-A″, yellow arrowheads in frames 5 and 6). At the same time, the AC protrusion retracted and the AC began to fuse with the utse, as indicated by diffusion of the *mCherry::moeABD* signal into adjacent uterine cells. The ring of adherens junctions subsequently expanded and the dorsal vulval lumen was opened in all control animals (Fig. 6A-A″, yellow arrowheads in frames 7-10; Movie 7). During these events, a distinct actin-rich domain remained localized at the interphase between vulF and the fusing AC in eight out of nine control animals (Fig. 6A′, yellow arrowheads in frames 6-8).

In *let-23AcKOEx* mutants, the AC protrusion did initially contact the vulF cells and bright HMR-1::GFP punctae also appeared in the AC, but the adherens junctions were disorganized and no clearly defined ring structure could be seen in eight out of 12 animals (Fig. 6B; Movie 8; Fig. S5B). Despite the absence of an appropriately organized adherens junction ring in the AC, the expansion of the dorsal vulval lumen did occur in *let-23AcKOEx* mutants, although it appeared to be delayed. To quantify the speed of dorsal lumen expansion, we measured the time from the last frame immediately before AC fusion to the point, when the adherens junctions started to expand (Δt) (e.g. Fig. 6A-A″, frames 4 and 8). The mean Δt was 2.69±0.91 h in control and 3.66±0.94 h in *let-23AcKOEx* animals (Fig. 6C).

In summary, time-lapse observation of vulval morphogenesis revealed that LET-23 signaling is necessary to assemble the newly forming adherens junctions in the AC into a ring-shaped structure, such that the AC and the vulF toroid can be appropriately joined before the dorsal vulval lumen expands.

### LET-23 signaling establishes a ring-shaped actin scaffold at the AC-vulF interphase

During AC invasion, an actin ring is generated in the AC around the neck of the forming invasive protrusion (Naegeli et al., 2017). To further examine the shape of the actin cytoskeleton during the connection between the AC and vulF toroid, we recorded confocal images of the F-actin reporter *qyIs50* in ventrally oriented animals at the L4.0 and the L4.3 substages, shortly before the AC fuses to form the utse. (The animals were rotated by the introduction of a *rol-6(su1006)* transgene.)

At the L4.0 substage, this analysis revealed a clearly visible actin ring in 75% of control animals compared with 21% of *let-23AcKO* animals (Fig. 6D,D′,F; Fig. S6A,B). At the L4.3 substage, we found that 68% of control animals had a distinct, actin-rich ring around the contact site between the ventral AC membrane and the dorsal surface of the vulF toroid (Fig. 6E,F; Fig. S6C), whereas, in 74% of *let-23AcKO* mutants, this actin-rich structure was either absent or barely recognizable (Fig. 6E′,F; Fig. S6D).

Thus, LET-23 signaling is necessary to reorganize the AC cytoskeleton and assemble an actin-rich scaffold, which may serve as a template to organize the newly forming adherens junctions at the AC-vulF contact site.

## DISCUSSION

### Microfluidic *C. elegans* handling

Both the previously introduced long-term image devices (Berger et al., 2021) and the short-term imaging variant introduced here provide a number of previously unknown possibilities. As described by Berger et al. (2021), the long-term imaging devices allow the observation of various processes occurring over multiple developmental stages within the same animal, resulting in a better understanding of developmental dynamics. The short-term devices introduced here complement this ability to study single animals over time with the ability to image large numbers of worms quickly at considerably higher throughput than possible using traditional agar-pad immobilization, without affecting worm viability or image quality. Devices for any developmental stage may be created following the simple design parameters outlined here, streamlining routine image acquisition as well as allowing the capture of subtle phenotypic variations only visible in large populations or at specific developmental stages. In the future, the short-term imaging devices may be combined with automated image acquisition tools for a variety of forward and reverse genetic screening experiments.

### LET-23 EGFR acts in the AC during early vulval morphogenesis

The nematode *C. elegans* has served as a powerful *in vivo* model to dissect the various roles of the EGFR signaling pathway during animal development and adulthood (Chamberlin and Sternberg, 1994; Clandinin et al., 1998; Konietzka et al., 2020; Pu et al., 2016; Van Buskirk and Sternberg, 2007). In particular, EGFR signaling is essential during the development of the *C. elegans* vulva, the egg-laying organ of the hermaphrodite. Here, we provide evidence for a previously unknown role of EGFR signaling in the uterine AC during invasion and vulval morphogenesis. Using a conditional *let-23* KO allele, we found that inactivation of LET-23 EGFR in the AC and ventral uterine cells results in a defective interconnection between the vulval toroids and the ventral uterus. Even though the FLP driver line used is not specific to the AC, because it also induced recombination in the adjacent VU cell, the defects we observed are most likely the result of the loss of LET-23 EGFR activity in the AC, because it is the only cell in the ventral uterus expressing LET-23 during vulval invagination and toroid formation. Only at a later stage could LET-23 expression be detected in the four uv1 cells that are specified during the mid-L4 stage by an EGF signal from the vulF cells (Chang et al., 1999). The uv1 cells join with the vulF toroid and utse only after the AC has connected with the vulF and fused to form the utse; animals lacking uv1 cells develop a normal vulval-uterine connection (Ihara et al., 2011; Johnson et al., 2009). Therefore, it is unlikely that the earlier defects in AC positioning and AC-vulF junction formation observed after inactivation of LET-23 are caused by a defect in uv1 fate specification or by undetectable LET-23 expression in other uterine cells. However, we cannot exclude the possibility that some of the defects observed later during dorsal lumen morphogenesis, such as the delay in dorsal lumen opening, may be caused by a lack of uv1 cells.

### Reciprocal EGFR signaling positions the AC and promotes BM breaching

**Fig. 7.**
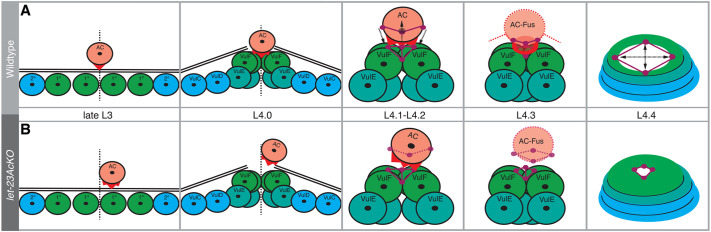
**LET-23 signaling in the AC controls formation of the uterine-vulval connection.** (A) The AC connects the ventral uterus to the vulval toroids. This process involves AC alignments and invasion at the vulval midline (L3 to L4.0) and formation of ring-shaped adherens junctions at the AC-vulF contact site (L4.1 to L4.2), allowing dorsal lumen expansion (L4.3 to L4.4). (B) Inactivation of LET-23 EGFR in the AC perturbs AC alignment, adherens junction organization and dorsal lumen opening. Colours represent the AC (orange), primary VPC lineage (greens), secondary VPC lineage (blue), adherens junctions (violet), cytoskeleton components (red) and BM (black).

After inactivation of LET-23 EGFR in the AC, we observed a mispositioning of the AC at the vulval midline. During vulval induction in L2 larvae, polarized LIN-3 secretion from the AC acts as an attractive cue for the VPCs (Grimbert et al., 2016; Mereu et al., 2020), with the closest VPC (P6.p) migrating underneath the AC and adopting the primary cell fate. At the same time, P6.p produces the neuropeptide-like ligand NLP-26 which creates a positive feedback by further polarizing LIN-3 secretion towards P6.p (Mereu et al., 2020). Our results suggest that after the initial positioning of the AC relative to the VPCs has been achieved during cell fate specification, LIN-3 secreted by the primary descendants of P6.p stabilizes the AC at the vulval midline by activating LET-23 EGFR signaling in the AC (Fig. 7). LIN-3 expression has been detected in the vulF cells from the L4.0 stage onward (Saffer et al., 2011), and a recent study found expression of EGL-38, the transcription factor inducing *lin-3* expression in the primary vulval cells, as early as at the Pn.px stage in mid-L3 larvae (Rajakumar and Chamberlin, 2007; Webb Chasser et al., 2019). Together, these findings suggest that a reciprocal LIN-3 signal from the primary vulval cells, originally identified by Chang et al. (1999), may act already before the onset of vulval lumen formation as a guidance cue for the AC. This hypothesis is consistent with our observation that inactivation of LET-23 in the AC results in the formation of smaller invasive protrusions, a slight delay in BM breaching and an enhancement of the invasion defect caused by loss of *unc-6 netrin(lf)* function (Ziel et al., 2009). The comparably weaker enhancement of the *unc-40(lf)* AC invasion defects observed after LET-23 inactivation may be explained by the fact that, in contrast to *unc-6(lf)*, the actin cytoskeleton in *unc-40(lf)* mutants remains polarized at the invasive AC membrane (Wang et al., 2014).

Sherwood and Sternberg (2003) have previously postulated the existence of one or multiple additional invasion cues from the primary vulval cells, possibly sent as diffusible signals acting at a distance (Sherwood and Sternberg, 2003). Thus, we propose that LIN-3 may be one of several, redundant guidance factors expressed by the primary vulval cells. If the AC is not correctly localized at the vulval midline prior to invasion, it cannot focus its actin-rich invadopodia at the mid-point between the primary vulF cells, which may explain the reduced efficiency in BM breaching (Hagedorn et al., 2014). Accordingly, global inactivation of *lin-3 egf* by RNAi not only causes a vulvaless phenotype, but also results in a reduced number, slower assembly and longer life time of the AC invadopodia (Lohmer et al., 2016). Given that the BMs were breached in around 40% of *let-23AcKO; unc-6(ev400lf)* double mutants at a later stage (L4.4), additional guidance cues besides LIN-3 EGF and UNC-6 Netrin may exist to promote BM breaching by the AC.

### LET-23 signaling is necessary to connect the vulva to the uterus

After breaching the BMs at the vulval midline, the AC organizes the formation of the dorsal vulval lumen (Estes and Hanna-Rose, 2009). The AC needs to occupy the space at the vulval midline physically, where the dorsal lumen will form, until it fuses with the uterine seam cell to stabilize the vulval-uterine connection (Estes and Hanna-Rose, 2009; Sapir et al., 2007).

Using microfluidic-based long-term imaging (Berger et al., 2021), we could for the first time continuously capture the whole process of vulval morphogenesis from VPC invagination until eversion within the same animal. This in-depth look into organogenesis revealed that the AC induces a complex rearrangement of newly forming adherens junctions that first constrict and then expand at the AC-vulF contact site (Fig. 7). Our data further suggest that LET-23 signaling in the AC organizes the formation of a circular actin ring, which may provide a scaffold for the formation of the connection between the uterus and vulva. The ring-shaped adherens junctions at the AC-vulF contact site may be necessary to expand the dorsal vulval lumen subsequently, and allow other uterine cells, such as the uv1 cells, to connect to the dorsal vulval toroids. However, the delayed expansion of the dorsal lumen observed after inactivation of LET-23 may also be due to defects in the specification of the uv1 cells.

Numerous studies of developmental processes have demonstrated that signaling by the EGFR or other RTKs reorganizes cell junctions and the connecting actomyosin cytoskeleton. For example, activation of VEGF signaling in endothelial cells leads to the phosphorylation of VE-cadherin, which reorganizes the adherens junction and dissociates the α/β-catenin complex that normally anchors the actin cytoskeleton to adherens junctions, resulting in increased endothelial permeability and enhanced transendothelial migration of leukocytes (Gavard et al., 2008; Sidibé and Imhof, 2014). Moreover, EGFR-dependent PI3K activation after mechanical stress promotes cell contractility mediated by the formation of new focal adhesions and ROCK-dependent activation of myosin II (Muhamed et al., 2016), and EGFR signaling during ommatidia morphogenesis in *Drosophila* is necessary for myosin II-dependent apical junctional remodeling (Robertson et al., 2012). Therefore, LET-23 EGFR signaling in the AC may play a similar role by reorganizing the adherens junctions at the AC-vulF interphase and by controlling actomyosin-mediated cell shape changes.

In conclusion, our data indicate that LET-23 EGFR signaling in the AC is necessary to align the developing vulva to the uterus and organize newly forming adherens junctions connecting the two developing organs.

## MATERIALS AND METHODS

### Worm maintenance

All strains used in this study were grown under standard conditions as described previously (Brenner, 1974). Animals were maintained on NGM plates [produced in house following standard protocols: 17 g Agar, 3 g NaCl, 2.5 g Peptone, 1 ml 1 M CaCl_2_, 1 ml 1 M MgSO_4_, 25 ml KPO Buffer (108.3 g KH_2_PO_4_, 35.6 g K_2_HPO_4_, H_2_O to 1 l), 1 ml cholesterol (5 mg/ml in ethanol), H_2_O to 1 l; sterilized by autoclaving; all chemicals from Sigma-Aldrich] seeded with OP50 *Escherichia coli* bacteria as a food source at 20°C, unless noted otherwise. The N2 Bristol strain was used as wild type. The following alleles and transgenes were used in this study:

LGI: *hmr-1(cp21[hmr-1::gfp+LoxP])* (Marston et al., 2016), *unc-40(e271)* (Brenner, 1974).

LGII: *let-23(zh131[FRT::let-23::FRT::GFP::LoxP::FLAG::let-23])* (Konietzka et al., 2020), *bqSi294[P_hsp16.41_>FRT::mCherry::his-58::FRT::GFP::his-58, unc-119(+)]* (Muñoz-Jiménez et al., 2017).

LGIII: *zhIs146[P_ACEL-pes-10_>2xNLS-FLP-D5, unc-119(+)]* (this study), *unc-119(ed3)* (Maduro and Pilgrim, 1995), *oxTi444[ttTi5605+NeoR(+)+unc18(+)]* (Frøkjær-Jensen et al., 2014)*.*

LGIV: *qyIs10[P_lam-1_>lam-1::GFP*, *unc-119(+)]* (Ziel et al., 2009).

LGV: *qyIs50[P_cdh-3_>mCherry::moeABD, unc-119(+)]* (Ziel et al., 2009), *qyIs127[P_lam-1_>lam-1::mCherry, unc-119(+)]* (Ihara et al., 2011).

LGX: *unc-6(ev400)* (Hedgecock et al., 1990), *qyIs24 [P_cdh-3_>mCherry::PLCδ^PH^, unc119(+)]* (Ziel et al., 2009), extrachromosomal array: *zhEx614[P_ACEL-pes-10_>2xNLS-FLP-D5, P_myo-2_>mCherry]* (this study), *zhEx683-684[rol-6(1006); Pmyo-2::mCherry]* (this study)*.*

See Table S1 for genotypes of all strains used in this study.

### Microfluidic device fabrication

Short-term imaging devices were fabricated following standard soft- and photolithographic protocols (Xia and Whitesides, 1998). Master molds were fabricated on silicon wafers (Si-Wafer 4P0/>1/525±25/SSP/TTV<10, Siegert Wafer) using SU8 photoresist (GM1050 and GM1060, Gersteltec). Layers of different height were fabricated on the same wafer and aligned to each other using a mask aligner (UV-KUB3, Kloe). Masters for the L3s devices were fabricated with heights of 5 and 20 μm (Supplementary files 1 and 3), and for the L4s devices at heights of 5 and 22 μm (Supplementary files 2 and 3). All wafers were treated with chlorotrimethyl silane (Sigma-Aldrich) before PDMS casting, to prevent PDMS adhesion. Devices were fabricated from a single ∼4 mm layer of PDMS (Elastosil RT601; ratio 10:1, Wacker) and cured at 70°C for at least 1 h. Once hardened, the device was carefully removed from the wafer, cut to size, access holes punched (15G Catheter Punch, Syneo) and the device bonded to a cover glass (75×25 mm cover glass with selected thickness 0.17±0.01 mm, Hecht Assistant) using air plasma (Zepto Plasma cleaner, Diener). For detailed information on device fabrication, see Supplementary Materials and Methods, ‘Device fabrication – short-term imaging’. Long-term imaging devices were produced as described by Berger et al. (2021).

### Microfluidic device operation

For short-term imaging experiments, worms were grown on NGM plates as described above until the desired age. Animals were washed off the plate using S-Basal buffer [5.85 g NaCl, 1 g K_2_HPO_4_, 6 gKH_2_PO, 1 ml cholesterol (5 mg/ml in ethanol), H_2_O to 1 L; sterilized by autoclaving; Sigma-Aldrich] supplemented with 1 wt% Pluronic F127 (Sigma-Aldrich), and washed twice with fresh S-Basal+F127 to remove any debris. Worms were loaded into the short-term device using a 1 ml syringe filled with S-Basal+F127, attached to a short piece of 1/16″ Tygon tubing (15157044, Thermo Fisher Scientific) using a blunt needle (300-35-970, 23G blunt needle, Distrelec). Washed worms were gently sucked into the tubing, which was then attached to the device inlet. Animals were loaded by applying pressure on the syringe plunger and the process was observed under a dissection microscope (MZ12.5, Leica). Once all channels were filled, the tubing was removed and, if needed, the device was flushed with anesthetic solution (100 mM tetramisole hydrochloride, Sigma-Aldrich). Finally, the device was mounted under a microscope (see following section) and images acquired as described in the following section. For detailed information on worm preparation and device operation, see Supplementary Materials and Methods, ‘Worm preparation’ and ‘Worm loading’. Long-term imaging devices were set up and operated as described by Berger et al. (2021).

### Image acquisition

All images were acquired on an upright/inverted microscope (either a BX61, Olympus, Switzerland or Ti-U, Nikon), equipped with a fluorescence light source (either UHP-T-460-DI and UHP-T-560-DI, Prizmatix or LedHUB, Omicron Laserage Laserprodukt GmbH), a brightfield LED (either pE-100wht, Coolled or MCWHLP1, Thorlabs) and a camera (iXon Ultra 888, Andor Oxford Instruments or Prime95B, Photometrics). *z*-stacks were acquired using a piezo objective drive (either Nano-F100, Mad City Labs or MIPOS 100 SG, Piezosystems). For confocal imaging, a spinning disk unit (XLight V2, Crest) was attached to the Olympus microscope, with the fluorescence LEDs coupled into the system via a 5 mm liquid light guide (LLG). Image acquisition was controlled using custom-built MATLAB scripts (MATLAB 2019b, MathWorks) and custom-built microcontrollers (Arduino Mega 2560) for coordination of fluorescence and brightfield LEDs, piezo and camera.

Images were acquired using a 60× water immersion lens (CFI Plan Apo VC 60×C WI NA1.2, Nikon), a 60× oil immersion lens (UPlanAPO 60×/1.40 Oil, Olympus), a CFI Plan Apo Lambda 60× Oil NA1.4, Nikon) or a 100× oil immersion lens (UPlanSAPO 100×/1.40 Oil, Olympus). All images were acquired at 20±0.5°C, with temperature controlled by the room air conditioning system.

### Image preprocessing and deconvolution

For analysis, the images of multiple parallel acquired worms were first cropped to the vulval region using a custom-built MATLAB script and sorted according to their developmental stages using DIC optics or fluorescent reporter signals, such as the *qyIs50[P_cdh-3_>mCherry]* AC marker (Ziel et al., 2009), as landmarks. Fluorescent images were processed with the Huygens deconvolution software (SVI, Centre for Microscopy and Image Analysis, University of Zürich) or, where indicated, using the YacuDecu implementation of CUDA-based Richardson Lucy deconvolution in MATLAB.

### Determination of developmental stages

Developmental substages of L4 animals (L4.0-L4.4) were assigned using morphological features observed with DIC microscopy, as described in Mok et al. (2015) and by assessing fluorescent reporters, such as *hmr-1::gfp(cp21), qyIs50[P_cdh-3_>mCherry::moeABD]* and *FRT::let-23::FRT::gfp(zh131)*.

### Quantification of vulval induction

Vulval induction was scored by counting the number of primary or secondary induced VPCs at the L4 stage using DIC microscopy. The vulval induction index VI was calculated by dividing the total number of induced VPCs by the number of animals scored, as described by Dutt et al. (2004) and Sternberg and Horvitz (1986).

### Quantification of AC positioning and polarity

Mid-sagittal planes of the adherens junction marker HMR-1::GFP (*cp21*) (Marston et al., 2016) were used to identify the selected landmarks shown as red, orange and magenta asterisks in Fig. 3C. AC to mid-vulF alignment was quantified with a semi-automated Fiji script (Schindelin et al., 2012) as follows: the AC mid-point along the AP axis (blue asterisk in Fig. 3C) was calculated as the middle between the two HMR-1::GFP punctae delineating the AC edges (orange asterisks in Fig. 3C). The relative alignment ratio R_A_ and the absolute AC-to-mid-vulF distance Δ in µm were calculated as illustrated (Fig. 3C). R_A_ was obtained by dividing distance *x* [between the calculated AC mid-point (blue asterisk in Fig. 3C) and the nearest vulA junction (either vulA1 or vulA2, red asterisks in Fig. 3C)] by distance *y* [between vulA1 and vulA2]. Δ refers to the absolute distance in µm along the AP axis between the AC mid-point (blue asterisk in Fig. 3C) and the mid-point between the vulF cells (magenta asterisk in Fig. 3C). For additional information, see the Fiji script ‘Analysis Script: AC alignment’ in the Supplementary information.

Dorsoventral AC polarity was measured in summed *z*-projections of the *qyIs50[P_cdh-3_>mcherry::moeABD]* reporter signal, as described by Mereu et al. (2020).

### Scoring AC invasion

BM breaching was scored in mid-L3 (Pn.px, Pn.pxx), early (L4.0) and mid-L4 (L4.4) larvae, as indicated in Fig. 4. The continuity of the BM was evaluated by fluorescence microscopy using either the *qyIs10[P_lam-1_>lam-1::GFP]* or the *qyIs127[P_lam-1_>lam-1::mCherry]* transgene.

### Quantification of AC protrusions

Pn.px-staged animals were loaded into the long-term imaging device (type L2-A, Berger et al., 2021), and confocal *z*-stacks (spacing 0.5 µm) of the *qyIs24 [P_cdh-3_>mCherry::PLCδ^PH^]* AC reporter as well as epifluorescence images of the *qyIs10[P_lam-1_>lam-1::GFP]* BM reporter were acquired every 5 min. Up to five animals were imaged in parallel at 60× magnification. Acquired images were deconvolved using the YacuDecu implementation of CUDA-based Richardson Lucy deconvolution before further processing. Deconvolved files were cropped approximately to the desired region of interest and registration using the StackReg and TurobReg Fiji plugins (Thévenaz et al., 1998). AC volume was then determined using a method similar to that described by Kelley et al. (2017). Images of the *qyIs24 [P_cdh-3_>mCherry::PLCδ^PH^]* channel were binarized with a constant threshold of 0.05 and overlaid with the *qyIs10[P_lam-1_>lam-1::GFP]* channel. The protrusion (the signal below the BM barrier upon first appearance of a BM breach) was then identified manually for each frame, and the pixels of the cropped protrusions were counted to calculate the protrusion volume (volume in µm^3^=total number of counted pixels×pixel width×pixel height×pixel depth). The mean of the calculated volumes for all measured control and *let-23AcKO* animals over a 90 min period is shown in Fig. 3H as red- and blue-dashed lines, respectively, along with ±1 s.d. (faded colors). Individual plots for each analyzed animal are shown in Fig. S3B,C. Illustrative 3D renderings of the cropped protrusions in Fig. 3F,G were generated using Fiji 3D viewer.

### Quantification of AC dynamics

L4.0-stage animals were loaded into the short-term imaging device (see Supplementary Materials and Methods, ‘Worm preparation’ and ‘Worm loading’), and *z*-stacks (spacing 0.25 µm) of the *qyIs50[P_cdh-3_>mcherry::moeABD]* reporter outlining the AC were acquired every 30 s for a total of 10 min. Up to three animals were imaged in parallel at 60× magnification. Acquired images were first deconvolved, cropped approximately to the required area and registered using the StackReg and TurobReg Fiji plugins (Thévenaz et al., 1998), followed by maximum intensity projection. In a second step, images were centered on the brightest point found in the AC, further cropped to a 144×90 pixel image and thresholded, yielding a binary image outlining the AC. Using the Fiji-based Image CorrelationJ plugin (Chinga and Syverud, 2007), the correlation indices for consecutive time points in a series were calculated to assess the degree by which the AC shape changed over time. The correlation index C_I_, used as a measure of AC dynamics, was calculated as the mean of all correlation indices in a time series.

### Long-term imaging vulval morphogenesis

Animals carrying the *hmr-1::gfp(cp21)* and *qyIs50[P_cdh-3_>mcherry::moeABD]* reporters were trapped in the long-term imaging devices (type L2-A, Berger et al., 2021) and *z*-stacks (spacing 0.25 µm) were acquired every 15 min for a total of 48 h from the early L3 stage until the L4.9 substage (vulval eversion). After cropping, *z*-stacks of each time point were registered using the StackReg and TurobReg Fiji plugins (Thévenaz et al., 1998) to correct for possible motion during image acquisition, deconvolved and then manually cropped around the vulval region. Each registered and cropped time point was 3D projected (*x*-, *y*- and *z*-projections) and the time points were concatenated to generate Movies 5-8.

### Imaging the actin cytoskeleton in the AC

An *Ex(rol-6(su1006)* transgene was introduced into animals carrying the *qyIs50[P_cdh-3_>mcherry::moeABD]* actin and the *hmr-1::gfp(cp21)* adherens junction markers to reorient them ventrally or dorsally in the microfluidic devices. Confocal images of ventrally oriented worms were acquired at the L4.0 and L4.3 substages, deconvolved and cropped to visualize the ring-shaped actin cytoskeleton at the AC-vulF interphase. In L4.0 substage animals, 12 out of 16 control (75%), and eight out of 38 *let-23AcKO* animals (21%) showed either a clearly defined continuous actin ring (Fig. S6AW1) or a semicircle (Fig. S6AW7), whereas the remaining control and *let-23AcKO* animals showed either barely distinguishable, disorganized rings (Fig. S6BW4) or no actin ring at all (Fig. S6BW8). In L4.3 substage animals, 20 out of 29 control (69%) and 11 out of 43 *let-23AcKO* animals (26%) showed either a clearly defined continuous actin ring (Fig. S6CW1) or a semicircle (Fig. S6CW4), whereas the remaining control and *let-23AcKO* animals showed either barely distinguishable, disorganized rings (Fig. S6DW4) or no actin ring at all (Fig. S6W7). The fraction of animals containing either a complete or semicircle actin ring is shown in Fig. 6F.

### Plasmid construction and single-copy genome insertion

For plasmid pSS23(P*_ACEL-pes-10_::2xNLS-FLP-D5::let-858 3′UTR* in pCFJ151), the anchor cell-specific enhancer element of *lin-3* (P_ACEL_) (Hwang and Sternberg, 2004) coupled with the minimal *Δpes-*10 promoter element was amplified from pMW87 with the primer pair OEH185/OEH187 and the 1.9 kb 2XNLS-FLP-D5::let-858 3′UTR fragment was amplified from pMLS262 (Schwartz and Jorgensen, 2016) with the primer pair OEH188/OJE111. These two fragments were subcloned and the P*_ACEL-pes-10_::2xNLS-FLP-D5::let-858 3′UTR* fragment was subsequently amplified with primer pair OSS200/OSS202 and integrated into pCJF151, after digestion with AvrII/SpeI (Frøkjær-Jensen et al., 2014), by Gibson assembly cloning (Gibson et al., 2009). pSS23 was used to generate a single-copy insertion in *oxTi444[ttTi5605+NeoR(+)+unc18(+)]; unc-119(ed3)* on LGIII using the MosSci protocol (Frøkjær-Jensen et al., 2014). Table S2 contains a list of the primers used in this study.

### Statistical analysis

Statistical analysis of continuous measurements (R_A_, Δ, I_DV_ and C_I_) was performed using the Student's *t*-test. To analyze the difference between the range of the values, an F-test for variance was performed for (Δ). For quantifications of discrete values (BM breaching and actin ring formation), a nonparametric Mann–Whitney *U* test was used, as indicated in the figure legends. Prism 9.0.0 (Graphpad) software was used for data analysis and plotting.

## Supplementary Material

Supplementary information

Reviewer comments

## References

[DEV199900C1] Aroian, R. V., Koga, M., Mendel, J. E., Ohshima, Y. and Sternberg, P. W. (1990). The let-23 gene necessary for Caenorhabditis elegans vulval induction encodes a tyrosine kinase of the EGF receptor subfamily. *Nature* 348, 693-699. 10.1038/348693a0 1979659

[DEV199900C2] Berger, S., Spiri, S., deMello, A. and Hajnal, A. (2021). Microfluidic-based imaging of complete Caenorhabditis elegans larval development. *Development* 148, dev199674. 10.1242/dev.199674 34170296 PMC8327290

[DEV199900C3] Berset, T., Hoier, E. F., Battu, G., Canevascini, S. and Hajnal, A. (2001). Notch inhibition of RAS signaling through MAP kinase phosphatase LIP-1 during *C. elegans* vulval development. *Science* 291, 1055-1058. 10.1126/science.1055642 11161219

[DEV199900C4] Brenner, S. (1974). The genetics of Caenorhabditis elegans. *Genetics* 77, 71-94. 10.1093/genetics/77.1.71 4366476 PMC1213120

[DEV199900C5] Chamberlin, H. M. and Sternberg, P. W. (1994). The lin-3/let-23 pathway mediates inductive signalling during male spicule development in Caenorhabditis elegans. *Development* 120, 2713-2721. 10.1242/dev.120.10.2713 7607066

[DEV199900C6] Chang, C., Newman, A. P. and Sternberg, P. W. (1999). Reciprocal EGF signaling back to the uterus from the induced *C. elegans* vulva coordinates morphogenesis of epithelia. *Curr. Biol.* 9, 237-246. 10.1016/S0960-9822(99)80112-2 10074449

[DEV199900C7] Chen, J., Zeng, F., Forrester, S. J., Eguchi, S., Zhang, M.-Z. and Harris, R. C. (2016). Expression and function of the epidermal growth factor receptor in physiology and disease. *Physiol. Rev.* 96, 1025-1069. 10.1152/physrev.00030.2015 33003261

[DEV199900C8] Chinga, G. and Syverud, K. (2007). Quantification of paper mass distributions within local picking areas. *Nordic Pulp Paper Res. J.* 22, 441-446. 10.3183/npprj-2007-22-04-p441-446

[DEV199900C9] Chung, K., Crane, M. M. and Lu, H. (2008). Automated on-chip rapid microscopy, phenotyping and sorting of C. elegans. *Nat. Meth.* 5, 637-643. 10.1038/nmeth.1227 18568029

[DEV199900C10] Clandinin, T. R., DeModena, J. A. and Sternberg, P. W. (1998). Inositol trisphosphate mediates a RAS-independent response to LET-23 receptor tyrosine kinase activation in *C. elegans*. *Cell* 92, 523-533. 10.1016/S0092-8674(00)80945-9 9491893

[DEV199900C11] Dutt, A., Canevascini, S., Froehli-Hoier, E. and Hajnal, A. (2004). EGF signal propagation during *C. elegans* vulval development mediated by ROM-1 rhomboid. *PLoS Biol.* 2, e334. 10.1371/journal.pbio.0020334 15455032 PMC519001

[DEV199900C12] Estes, K. A. and Hanna-Rose, W. (2009). The anchor cell initiates dorsal lumen formation during *C. elegans* vulval tubulogenesis. *Dev. Biol.* 328, 297-304. 10.1016/j.ydbio.2009.01.034 19389356

[DEV199900C13] Farooqui, S., Pellegrino, M. W., Rimann, I., Morf, M. K., Müller, L., Fröhli, E. and Hajnal, A. (2012). Coordinated lumen contraction and expansion during vulval tube morphogenesis in Caenorhabditis elegans. *Dev. Cell* 23, 494-506. 10.1016/j.devcel.2012.06.019 22975323

[DEV199900C14] Frøkjær-Jensen, C., Wayne Davis, M., Hopkins, C. E., Newman, B. J., Thummel, J. M., Olesen, S.-P., Grunnet, M. and Jorgensen, E. M. (2008). Single-copy insertion of transgenes in Caenorhabditis elegans. *Nat. Genet.* 40, 1375-1383. 10.1038/ng.248 18953339 PMC2749959

[DEV199900C15] Frøkjær-Jensen, C., Davis, M. W., Sarov, M., Taylor, J., Flibotte, S., LaBella, M., Pozniakovsky, A., Moerman, D. G. and Jorgensen, E. M. (2014). Random and targeted transgene insertion in Caenorhabditis elegans using a modified Mos1 transposon. *Nat. Meth.* 11, 529-534. 10.1038/nmeth.2889 PMC4126194 24820376

[DEV199900C16] Gauthier, K. D. and Rocheleau, C. E. (2021). Golgi localization of the LIN-2/7/10 complex points to a role in basolateral secretion of LET-23 EGFR in the Caenorhabditis elegans vulval precursor cells. *Development* 148, dev194167. 10.1242/dev.194167 33526581 PMC10692275

[DEV199900C17] Gavard, J., Patel, V. and Gutkind, J. S. (2008). Angiopoietin-1 prevents VEGF-induced endothelial permeability by sequestering Src through mDia. *Dev. Cell* 14, 25-36. 10.1016/j.devcel.2007.10.019 18194650

[DEV199900C18] Gibson, D. G., Young, L., Chuang, R.-Y., Venter, J. C., Hutchison, C. A. and Smith, H. O. (2009). Enzymatic assembly of DNA molecules up to several hundred kilobases. *Nat. Meth.* 6, 343-345. 10.1038/nmeth.1318 19363495

[DEV199900C19] Goswami, S., Sahai, E., Wyckoff, J. B., Cammer, M., Cox, D., Pixley, F. J., Stanley, E. R., Segall, J. E. and Condeelis, J. S. (2005). Macrophages promote the invasion of breast carcinoma cells via a colony-stimulating factor-1/epidermal growth factor paracrine loop. *Cancer Res.* 65, 5278-5283. 10.1158/0008-5472.CAN-04-1853 15958574

[DEV199900C20] Greenwald, I. S., Sternberg, P. W. and Horvitz, H. R. (1983). The lin-12 locus specifies cell fates in Caenorhabditis elegans. *Cell* 34, 435-444. 10.1016/0092-8674(83)90377-X 6616618

[DEV199900C21] Grimbert, S., Tietze, K., Barkoulas, M., Sternberg, P. W., Félix, M.-A. and Braendle, C. (2016). Anchor cell signaling and vulval precursor cell positioning establish a reproducible spatial context during *C. elegans* vulval induction. *Dev. Biol.* 416, 123-135. 10.1016/j.ydbio.2016.05.036 27288708

[DEV199900C22] Gupta, B. P., Hanna-Rose, W. and Sternberg, P. (2012). Morphogenesis of the vulva and the vulval-uterine connection. *WormBook*, 1-20. 10.1895/wormbook.1.152.1 10.1895/wormbook.1.152.1 PMC5402222 23208727

[DEV199900C23] Haag, A., Gutierrez, P., Bühler, A., Walser, M., Yang, Q., Langouët, M., Kradolfer, D., Fröhli, E., Herrmann, C. J., Hajnal, A. et al. (2014). An *in vivo* EGF receptor localization screen in *C. elegans* Identifies the Ezrin homolog ERM-1 as a temporal regulator of signaling. *PLoS Genet.* 10, e1004341. 10.1371/journal.pgen.1004341 24785082 PMC4006739

[DEV199900C24] Hagedorn, E. J., Kelley, L. C., Naegeli, K. M., Wang, Z., Chi, Q. and Sherwood, D. R. (2014). ADF/cofilin promotes invadopodial membrane recycling during cell invasion *in vivo*. *J. Cell Biol.* 204, 1209-1218. 10.1083/jcb.201312098 24662568 PMC3971745

[DEV199900C25] Hedgecock, E. M., Culotti, J. G. and Hall, D. H. (1990). The unc-5, unc-6, and unc-40 genes guide circumferential migrations of pioneer axons and mesodermal cells on the epidermis in *C. elegans*. *Neuron* 4, 61-85. 10.1016/0896-6273(90)90444-K 2310575

[DEV199900C26] Hill, R. J. and Sternberg, P. W. (1992). The gene lin-3 encodes an inductive signal for vulval development in *C. elegans*. *Nature* 358, 470-476. 10.1038/358470a0 1641037

[DEV199900C27] Hsu, J. L. and Hung, M.-C. (2016). The role of HER2, EGFR, and other receptor tyrosine kinases in breast cancer. *Cancer Metastasis Rev.* 35, 575-588. 10.1007/s10555-016-9649-6 27913999 PMC5215954

[DEV199900C28] Hwang, B. J. and Sternberg, P. W. (2004). A cell-specific enhancer that specifies lin-3 expression in the *C. elegans* anchor cell for vulval development. *Development* 131, 143-151. 10.1242/dev.00924 14660442

[DEV199900C29] Ihara, S., Hagedorn, E. J., Morrissey, M. A., Chi, Q., Motegi, F., Kramer, J. M. and Sherwood, D. R. (2011). Basement membrane sliding and targeted adhesion remodels tissue boundaries during uterine-vulval attachment in Caenorhabditis elegans. *Nat. Cell Biol.* 13, 641-651. 10.1038/ncb2233 21572423 PMC3107347

[DEV199900C30] Iwamoto, R. and Mekada, E. (2006). ErbB and HB-EGF signaling in heart development and function. *Cell Struct. Funct.* 31, 1-14. 10.1247/csf.31.1 16508205

[DEV199900C31] Johnson, R. W., Liu, L. Y., Hanna-Rose, W. and Chamberlin, H. M. (2009). The Caenorhabditis elegans heterochronic gene lin-14 coordinates temporal progression and maturation in the egg-laying system. *Dev. Dyn.* 238, 394-404. 10.1002/dvdy.21837 19161245

[DEV199900C32] Kelley, L. C., Wang, Z., Hagedorn, E. J., Wang, L., Shen, W., Lei, S., Johnson, S. A. and Sherwood, D. R. (2017). Live-cell confocal microscopy and quantitative 4D image analysis of anchor-cell invasion through the basement membrane in Caenorhabditis elegans. *Nat. Protoc.* 12, 2081-2096. 10.1038/nprot.2017.093 28880279 PMC5915360

[DEV199900C33] Konietzka, J., Fritz, M., Spiri, S., McWhirter, R., Leha, A., Palumbos, S., Costa, W. S., Oranth, A., Gottschalk, A., Miller, D. M. et al. (2020). Epidermal growth factor signaling promotes sleep through a combined series and parallel neural circuit. *Curr. Biol.* 30, 1-16.e13. 10.1016/j.cub.2019.10.048 31839447

[DEV199900C34] Lohmer, L. L., Clay, M. R., Naegeli, K. M., Chi, Q., Ziel, J. W., Hagedorn, E. J., Park, J. E., Jayadev, R. and Sherwood, D. R. (2016). A sensitized screen for genes promoting invadopodia function *in vivo*: CDC-42 and Rab GDI-1 direct distinct aspects of invadopodia formation. *PLoS Genet.* 12, e1005786. 10.1371/journal.pgen.1005786 26765257 PMC4713207

[DEV199900C35] Maduro, M. and Pilgrim, D. (1995). Identification and cloning of unc-119, a gene expressed in the Caenorhabditis elegans nervous system. *Genetics* 141, 977-988. 10.1093/genetics/141.3.977 8582641 PMC1206859

[DEV199900C36] Marston, D. J., Higgins, C. D., Peters, K. A., Cupp, T. D., Dickinson, D. J., Pani, A. M., Moore, R. P., Cox, A. H., Kiehart, D. P. and Goldstein, B. (2016). MRCK-1 drives apical constriction in *C. elegans* by linking developmental patterning to force generation. *Curr. Biol.* 26, 2079-2089. 10.1016/j.cub.2016.06.010 27451898 PMC4996705

[DEV199900C37] Mereu, L., Morf, M. K., Spiri, S., Gutierrez, P., Escobar-Restrepo, J. M., Daube, M., Walser, M. and Hajnal, A. (2020). Polarized epidermal growth factor secretion ensures robust vulval cell fate specification in Caenorhabditis elegans. *Development* 147, dev175760. 10.1242/dev.175760 32439759 PMC7286359

[DEV199900C38] Mok, D. Z. L., Sternberg, P. W. and Inoue, T. (2015). Morphologically defined sub-stages of *C. elegans* vulval development in the fourth larval stage. *BMC Dev. Biol.* 15, 26-28. 10.1186/s12861-015-0076-7 26066484 PMC4464634

[DEV199900C39] Mondal, S., Hegarty, E., Martin, C., Gökçe, S. K., Ghorashian, N. and Ben-Yakar, A. (2016). Large-scale microfluidics providing high-resolution and high-throughput screening of Caenorhabditis elegans poly-glutamine aggregation model. *Nat. Commun.* 7, 13023. 10.1038/ncomms13023 27725672 PMC5062571

[DEV199900C40] Muhamed, I., Wu, J., Sehgal, P., Kong, X., Tajik, A., Wang, N. and Leckband, D. E. (2016). E-cadherin-mediated force transduction signals regulate global cell mechanics. *J. Cell. Sci.* 129, 1843-1854. 10.1242/jcs.185447 26966187 PMC4893802

[DEV199900C41] Muñoz-Jiménez, C., Ayuso, C., Dobrzynska, A., Torres-Mendéz, A., Ruiz, P. de L. C. and Askjaer, P. (2017). An efficient FLP-based toolkit for spatiotemporal control of gene expression in Caenorhabditis elegans. *Genetics* 206, 1763-1778. 10.1534/genetics.117.201012 28646043 PMC5560786

[DEV199900C42] Naegeli, K. M., Hastie, E., Garde, A., Wang, Z., Keeley, D. P., Gordon, K. L., Pani, A. M., Kelley, L. C., Morrissey, M. A., Chi, Q. et al. (2017). Cell invasion in vivo via rapid exocytosis of a transient lysosome-derived membrane domain. *Dev. Cell* 43, 403-417.e10. 10.1016/j.devcel.2017.10.024 29161591 PMC5726793

[DEV199900C43] Pu, P., Stone, C. E., Burdick, J. T., Murray, J. I. and Sundaram, M. V. (2016). The Lipocalin LPR-1 cooperates with LIN-3/EGF signaling to maintain narrow tube integrity in Caenorhabditis elegans. *Genetics* 205, 1247-1260. 10.1534/genetics.116.195156 28040739 PMC5340336

[DEV199900C44] Rajakumar, V. and Chamberlin, H. M. (2007). The Pax2/5/8 gene egl-38 coordinates organogenesis of the *C. elegans* egg-laying system. *Dev. Biol.* 301, 240-253. 10.1016/j.ydbio.2006.08.068 17020758

[DEV199900C45] Rajaram, P., Chandra, P., Ticku, S., Pallavi, B. K., Rudresh, K. B. and Mansabdar, P. (2017). Epidermal growth factor receptor: Role in human cancer. *Indian J. Dent. Res.* 28, 687-694. 10.4103/ijdr.IJDR_534_16 29256471

[DEV199900C46] Robertson, F., Pinal, N., Fichelson, P. and Pichaud, F. (2012). Atonal and EGFR signalling orchestrate rok- and Drak-dependent adherens junction remodelling during ommatidia morphogenesis. *Development* 139, 3432-3441. 10.1242/dev.080762 22874916 PMC3424046

[DEV199900C47] Saffer, A. M., Kim, D. H., van Oudenaarden, A. and Horvitz, H. R. (2011). The Caenorhabditis elegans synthetic multivulva genes prevent ras pathway activation by tightly repressing global ectopic expression of lin-3 EGF. *PLoS Genet.* 7, e1002418-e11. 10.1371/journal.pgen.1002418 22242000 PMC3248470

[DEV199900C48] San-Miguel, A., Kurshan, P. T., Crane, M. M., Zhao, Y., McGrath, P. T., Shen, K. and Lu, H. (2016). Deep phenotyping unveils hidden traits and genetic relations in subtle mutants. *Nat. Comm.* 7, 12990. 10.1038/ncomms12990 PMC5122966 27876787

[DEV199900C49] Sapir, A., Choi, J., Leikina, E., Avinoam, O., Valansi, C., Chernomordik, L. V., Newman, A. P. and Podbilewicz, B. (2007). AFF-1, a FOS-1-regulated fusogen, mediates fusion of the anchor cell in *C. elegans*. *Dev. Cell* 12, 683-698. 10.1016/j.devcel.2007.03.003 17488621 PMC1975806

[DEV199900C50] Schindelin, J., Arganda-Carreras, I., Frise, E., Kaynig, V., Longair, M., Pietzsch, T., Preibisch, S., Rueden, C., Saalfeld, S., Schmid, B. et al. (2012). Fiji: an open-source platform for biological-image analysis. *Nat. Meth.* 9, 676-682. 10.1038/nmeth.2019 PMC3855844 22743772

[DEV199900C51] Schindler, A. J. and Sherwood, D. R. (2013). Morphogenesis of the caenorhabditis elegans vulva. *Wiley Interdiscip. Rev. Dev. Biol.* 2, 75-95. 10.1002/wdev.87 23418408 PMC3572792

[DEV199900C52] Schwartz, M. L. and Jorgensen, E. M. (2016). SapTrap, a toolkit for high-throughput CRISPR/Cas9 gene modification in Caenorhabditis elegans. *Genetics* 202, 1277-1288. 10.1534/genetics.115.184275 26837755 PMC4905529

[DEV199900C53] Segatto, O., Anastasi, S. and Alemà, S. (2011). Regulation of epidermal growth factor receptor signalling by inducible feedback inhibitors. *J. Cell. Sci.* 124, 1785-1793. 10.1242/jcs.083303 21576352

[DEV199900C54] Sharma-Kishore, R., White, J. G., Southgate, E. and Podbilewicz, B. (1999). Formation of the vulva in Caenorhabditis elegans: a paradigm for organogenesis. *Development* 126, 691-699. 10.1242/dev.126.4.691 9895317

[DEV199900C55] Sherwood, D. R. and Sternberg, P. W. (2003). Anchor Cell Invasion into the Vulval Epithelium in *C. elegans*. *Dev. Cell* 5, 21-31. 10.1016/S1534-5807(03)00168-0 12852849

[DEV199900C56] Sibilia, M., Kroismayr, R., Lichtenberger, B. M., Natarajan, A., Hecking, M. and Holcmann, M. (2007). The epidermal growth factor receptor: from development to tumorigenesis. *Differentiation* 75, 770-787. 10.1111/j.1432-0436.2007.00238.x 17999740

[DEV199900C57] Sidibé, A. and Imhof, B. A. (2014). VE-cadherin phosphorylation decides: vascular permeability or diapedesis. *Nat. Immunol.* 15, 215-217. 10.1038/ni.2825 24549064

[DEV199900C58] Sisto, M., Lorusso, L., Ingravallo, G. and Lisi, S. (2017). Exocrine gland morphogenesis: insights into the role of Amphiregulin from development to disease. *Arch. Immunol. Ther. Exp. (Warsz)* 65, 477-499. 10.1007/s00005-017-0478-2 28593345

[DEV199900C59] Sternberg, P. W. and Horvitz, H. R. (1986). Pattern formation during vulval development in C. elegans. *Cell* 44, 761-772. 10.1016/0092-8674(86)90842-1 3753901

[DEV199900C60] Sundaram, M. V. (2006). RTK/Ras/MAPK signaling. *WormBook*, 1-19. 10.1895/wormbook.1.80.1

[DEV199900C61] Thévenaz, P., Ruttimann, U. E. and Unser, M. (1998). A pyramid approach to subpixel registration based on intensity. *IEEE Trans. Image Process.* 7, 27-41. 10.1109/83.650848 18267377

[DEV199900C62] Van Buskirk, C. and Sternberg, P. W. (2007). Epidermal growth factor signaling induces behavioral quiescence in Caenorhabditis elegans. *Nat. Neurosci.* 10, 1300-1307. 10.1038/nn1981 17891142

[DEV199900C63] Wang, Z., Linden, L. M., Naegeli, K. M., Ziel, J. W., Chi, Q., Hagedorn, E. J., Savage, N. S. and Sherwood, D. R. (2014). UNC-6 (netrin) stabilizes oscillatory clustering of the UNC-40 (DCC) receptor to orient polarity. *J. Cell Biol.* 206, 619-633. 10.1083/jcb.201405026 25154398 PMC4151141

[DEV199900C64] Webb Chasser, A. M., Johnson, R. W. and Chamberlin, H. M. (2019). EGL-38/Pax coordinates development in the Caenhorhabditis elegans egg-laying system through EGF pathway dependent and independent functions. *Mech. Dev.* 159, 103566. 10.1016/j.mod.2019.103566 31398431 PMC6855382

[DEV199900C65] Wee, P. and Wang, Z. (2017). Epidermal growth factor receptor cell proliferation signaling pathways. *Cancers (Basel)* 9, 52. 10.3390/cancers9050052 PMC5447962 28513565

[DEV199900C66] Xia, Y. and Whitesides, G. M. (1998). Soft lithography. *Annu. Rev. Mater. Sci.* 28, 153-184. 10.1146/annurev.matsci.28.1.153

[DEV199900C67] Yamaguchi, H., Lorenz, M., Kempiak, S., Sarmiento, C., Coniglio, S., Symons, M., Segall, J., Eddy, R., Miki, H., Takenawa, T. et al. (2005). Molecular mechanisms of invadopodium formation: the role of the N-WASP-Arp2/3 complex pathway and cofilin. *J. Cell Biol.* 168, 441-452. 10.1083/jcb.200407076 15684033 PMC2171731

[DEV199900C68] Yarden, Y. and Sliwkowski, M. X. (2001). Untangling the ErbB signalling network. *Nat. Rev. Mol. Cell Biol.* 2, 127-137. 10.1038/35052073 11252954

[DEV199900C69] Yoo, A. S., Bais, C. and Greenwald, I. (2004). Crosstalk between the EGFR and LIN-12/Notch pathways in *C. elegans* vulval development. *Science* 303, 663-666. 10.1126/science.1091639 14752159

[DEV199900C70] Ziel, J. W., Hagedorn, E. J., Audhya, A. and Sherwood, D. R. (2009). UNC-6 (netrin) orients the invasive membrane of the anchor cell in *C. elegans*. *Nat. Cell Biol.* 11, 183-189. 10.1038/ncb1825 19098902 PMC2635427

