# Supplementary material for: Reciprocal EGFR signaling in the anchor cell ensures precise inter-organ connection during Caenorhabditis elegans vulval morphogenesis
Source: Development. 2022 Jan 4;149(1):dev199900. doi: 10.1242/dev.199900 (PMC8783044; doi:10.1242/dev.199900)
Supplement: Reviewer comments [file develop-149-199900_review_history.pdf]

Development

Reciprocal EGFR signaling in the anchor cell ensures precise inter-organ connection during *Caenorhabditis elegans* vulval morphogenesis

Silvan Spiri, Simon Berger, Louisa Mereu, Andrew DeMello and Alex Hajnal
DOI: 10.1242/dev.199900

Editor: Swathi Arur

Review timeline

Original submission:	14 June 2021
Editorial decision:	8 July 2021
First revision received:	9 November 2021
Accepted:	30 November 2021

Original submission

First decision letter

MS ID#: DEVELOP/2021/199900

MS TITLE: Reciprocal EGFR signaling in the Anchor Cell ensures precise inter-organ connection during *C. elegans* vulval morphogenesis

AUTHORS: Silvan Spiri, Simon Berger, Louisa Mereu, Andrew deMello, and Alex Hajnal

I have now received all the referees' reports on the above manuscript, and have reached a decision. As you will see, the referees express considerable interest in your work, but have some significant criticisms that will need to be addressed before we can consider publication. Specifically, reviewers 2 and 3 point out several concerns on the rigor of the core analysis performed in the study. Development takes rigor of methods and imaging analysis very seriously, and thus these concerns will need to be satisfactorily addressed. First, the core assay to assess invasion of the anchor cell is confusing in its description and the quality of the data acquired is low resolution. The assay is specific to the imaging conducted to assess the large protrusion of the AC during invasion which occurs on the dorsal-ventral axis. It is likely that the imaging as conducted with the XY projections is a limitation of the microfluidic device used in the study. However, to present careful rigorous analysis of the LET-23/LIN-3 turnover and formation of the basement membrane protrusions at this time in development and invasion of the AC, the assay will need to be conducted with confocal microscope based time-lapse imaging used in the field which images the dorsal-ventral axis and provides high resolution analysis. This is particularly important validation for not only the microfluidic device being used but is critical for the biological analysis being presented. Reviewers 2 and 3 point out several steps in the current analysis which are weak in detail and lack use of appropriate controls. The reviewers also point out key papers in the field which will need to be cited and the work integrated in the context of the work presented in the current study.If you are able to revise the manuscript along the lines suggested, which involves further experiments and in this case microscopy imaging, I will be happy to receive a revised version of the manuscript. Your revised paper will be re-reviewed by one or more of the original referees, and acceptance of your manuscript will depend on your addressing satisfactorily the reviewers' major concerns.

Please attend to all of the reviewers' comments and ensure that you clearly highlight all changes made in the revised manuscript. Please avoid using 'Tracked changes' in Word files as these are lost in PDF conversion. I should be grateful if you would also provide a point-by-point response detailing

© 2022. Published by The Company of Biologists under the terms of the Creative Commons Attribution License (https://creativecommons.org/licenses/by/4.0/).

how you have dealt with the points raised by the reviewers in the 'Response to Reviewers' box. If you do not agree with any of their criticisms or suggestions please explain clearly why this is so.

Reviewer 1

Advance summary and potential significance to field

During development of the *C. elegans* reproductive system, the uterine and vulva lumens must be connected through a process that involves the gonadal anchor cell (AC) invading through two basement membranes.
Previous work has described AC invadopodia and various mechanisms that control its formation and placement. One gap in understanding has been the mechanism(s) that direct spatial placement of invadopodia so that the AC penetrates in the correct place. Here, Spiri et al provide an answer to this question.

Spiri et al report for the first time expression of LET-23/EGFR in the gonadal AC and show a functional role for it there in aligning the AC to vulval cells for efficient basement membrane breaching and enlargement of the gap to join the vulva and uterus lumens. They show that a ring-shaped adherens junction forms between the AC and the vulval cells during alignment/breaching, and that LET-23 is required for its formation and for actin and junction dynamics associated with this appearance of this structure. Since LIN-3/EGF is expressed in the P6.p-derived vulva cells that are known to be important for breaching, this suggests a model whereby vulval LIN-3/EGF signals to AC LET-23/EGFR to control actin dynamics, invadopodia placement, and ring-shaped junction assembly.

Spiri et al also report on new, widely applicable, microfluidics device for short-term imaging of immobilized larvae. I predict this will be widely adopted. I can't wait to contact the authors to request a prototype and detailed directions for making it! I also encourage the authors to find a vendor to commercialize it.

C. elegans vulva development is a textbook example for how signaling controls different aspects of organ formation. The data in this manuscript will become part of this narrative that every review article, textbook entry, and class lecture on the topic will include. The results are clearly presented, the data appear robust.

My only substantive comment is that the introduction could better set up the major gap in knowledge that the data will end up filling. Currently, the intro talks about too many other things besides the issue at hand, and it is not until the Discussion that authors mention the early results of Sherwood et al which showed the (unexplained) requirement for P6.p and the ability of P6.p-derived signals to act at a distance.

On my list of "experiments the reviewer would have loved to see but which are not essential for the authors to do": Look at dig-1 mutants or repeat VPC laser ablations of Sherwood et al in the LET-23 AC-KO – prediction is that displacement or removal of P6.p-derived signals should not have any further impact.

Comments for the author

My only substantive comment is that the introduction could better set up the major gap in knowledge that the data will end up filling. Currently, the intro talks about too many other things besides the issue at hand, and it is not until the Discussion that authors mention the early results of Sherwood et al which showed the (unexplained) requirement for P6.p and the ability of P6.p-derived signals to act at a distance.
On my list of "experiments the reviewer would have loved to see but which are not essential for the authors to do": Look at dig-1 mutants or repeat VPC laser ablations of Sherwood et al in the LET-23 AC-KO – prediction is that displacement or removal of P6.p-derived signals should not have any further impact.

© 2022. Published by The Company of Biologists under the terms of the Creative Commons Attribution License (https://creativecommons.org/licenses/by/4.0/).

Reviewer 2

Advance summary and potential significance to field

In this manuscript Spiri et al examine the role of the EGFR, LET-23, in regulating *C. elegans* vulval morphogenesis. They describe and utilize an innovative microfluidics system for high-throughput imaging of L3 and L4 stage animals which should be useful for the community studying post-embryonic development. Notably the authors identify a key-role for LIN-3/LET-23 reciprocal signaling post-fate specification as part of the previously unknown vulval cue. Specifically, the authors describe a novel role for LET-23 function autonomously in the AC initially functioning in an epistatic relationship with Netrin/UNC-6/UNC-40 signaling as a guidance cue facilitating AC invasion, with FLP-mediated excision of endogenous LET-23 in the AC demonstrating a requirement for AC expression of LET-23 for several aspects of uterine-vulval morphogenesis, including proper AC alignment to the 1° fated VPCs, proper timing of BM breach, and proper organization of adherens junctions coordinated with the F-actin cytoskeleton in relation to the connection between the innermost vulF cells and the AC following invasion.

Comments for the author

In general, I am supportive of the publication of this manuscript but have several suggestions that might improve the manuscript as well as the incorporation of relevant literature that is missing from the current manuscript.

1. Perhaps the most important or relevant literature that needs to be incorporated throughout the manuscript is Naegeli et al. 2018 (doi.org/10.1016/j.devcel.2017.10.024). There are several important aspects of AC biology that were identified in this paper that are likely relevant to the current manuscript including:

a. Following BM breach the AC generates a large protrusion in the first 55 minutes following AC invasion, peaking at 30 minutes post-breach. Was this phase of AC invasion/morphogenesis captured during timelapse in this study and if so, is it affected by loss of LET-23 in the AC? It would be important/interesting to know if the multiple protrusions captured in Figure 3H in LET-23KO ACs are a result of a loss of the main protrusion or reflective of the AC continuing to generate invadopodia post-invasion rather than switching machinery to the lysosome-dependent large protrusion likely utilized to facilitate initial BM gap expansion.

b. Endogenous Netrin/UNC-6 localizes to the 1° VPCs and is important for the orientation of the main protrusion generated by the AC in an UNC-40-dependent manner.

c. If an invasive protrusion is generated in LET-23KO animals, are characterized aspects of the invasive protrusion still localized to it in the absence of LET-23, including the exocyst complex (SNAP-29).

d. In addition to the F-actin ring structure described in Figure 6 of this manuscript, there is an initial F-actin ring generated in the AC (see Fig. 6B of Naegeli et al 2018) during protrusion formation. Is this F-actin ring still generated in LET-23KO animals? Is the dystroglycan-mediated barrier that separates protrusion from apical-lateral AC plasma membrane intact of perturbed in LET-23KO animals?

2. Is it possible to trap animals oriented dorsal/ventral in the microfluidics device (which seems like it will be incredibly useful for the field!) – then rather than using xz reconstructions and losing most of the resolution in imaging, you would be able to image at high resolution in ventral-oriented animals. This would make visualization and quantification of the later steps of vulval morphogenesis much easier. If the microfluidics device is incapable of capturing animals in this orientation, one can use coverslips to roll animals to a ventral orientation to capture these dynamics at high-resolution on spinning-disc confocal or even inject rol-6 or utilize non-excised SEC-animals which ~25% of the time will be ventral up on a silde.

Minor comments:

1. Page 3: avoid terms like “higher” and “lower” organisms – could use vertebrate vs invertebrates, but all of these organisms are extant and at the tips of their respective evolutionary lineage.

2. Figure 3: Consider using a red/green color-blind friendly heat map instead of the one used in Figure H’/G’.

© 2022. Published by The Company of Biologists under the terms of the Creative Commons Attribution License (https://creativecommons.org/licenses/by/4.0/).

Reviewer 3

Advance summary and potential significance to field

The manuscript entitled “Reciprocal EGFR signaling in the Anchor Cell ensures precise inter-organ connection during *C. elegans* vulval morphogenesis” (DEVELOP-2021-199900v1) describes the consequences of conditional deletion of the let-23 locus, which encodes the *C. elegans* EGFR-like protein, in the anchor cell (AC). This experiment led to the interesting observation of mis-positioning of the AC relative to the developing vulva and defects in the fusion of the two. This result is interesting because it represents a reciprocal signal of the developing primary lineage in the vulva back to the AC, which is the source of LIN-3/EGF that initially patterns the cell fates of the vulva, playing on theme of temporal iteration of the same signaling modules during development, the latter ones masked by the necessity of the earlier. Of additional interest is the finding that that the LET-23 signal in the AC proposed to govern invasion through the basement membrane is partially redundant with the Netrin signal originally described by the Sherwood lab. This work contributes to our understanding of developmental processes that refine precisely coordinated invasive cell movements during organogenesis, as well as insights into developmental homeostasis and/or fidelity: how organisms account for positional variation or size differences to still generate proper forms.

Comments for the author

Major concerns

- Regarding the main assay of the centering of the AC on vulval lineages: this is done by imaging of tagged HMR-1/beta-catenin to visualize adherens junctions of vulval lineages and an actin marker to visualize the AC. But how is the center point of each defined? We learn from the methods that this measurement is performed using a “semi-automated” process governed by a Fiji script. But we do not receive more information on this measurement, which underpins the most central assay of the paper. By visual inspection this assay is dubious: looking at the figures provided, it is unclear to this reviewer how the “center” of the AC or stack of toroids in the developing vulval could be defined. In some the placement of the “center of gravity” seems entirely subjective. Would these points in the developing animal be better defined by DIC for nuclei and the lumen of the invaginating pre-vulva? Or perhaps a combination of DIC and fluorescence?
  - o Given the centrality of this assay to the conclusions of this study, it behooves the authors to better define how these numbers are determined. This assay lies at the core of this manuscript and the analysis that flows from the assay. If a user is arbitrarily defining those midpoints in an assay with relatively small difference between experimental and control conditions, it seems like a source of subjectivity in the data analysis.
  - o I looked for notation that these values were assigned in blind assays, but did not find them. But looking at Figure 3, the slightest bit of subjectivity could result in the entire difference we see. I would have much higher confidence if images had been coded and scored blindly.
  - o So while the methods section says this process is semi-automated using Fiji, either the quantification process needs to be better documented or the data re-scored by a blind scorer. Any manual scoring with knowledge of genotypes could fatally skew the data.
- Also, is there a negative control assay for the FLP expression? Maybe the let-23AcKO but without FLP expression? Ectopic promoters or proteins are frequently sufficient to alter cell behavior somewhat, though perhaps less likely because zhIs146 is a single copy transgene. So what we are seeing would not necessarily due to deletion of LET-23, but rather could be ectopic expression of PACEL-pes-10 (the promoter) disrupting the system. Perhaps the key control here would be the effects of zhIs146 on centering of vulva and AC in the absence of the let-23AcKO construct, thus showing that the effect depends on deletion of let-23 sequences. Or not!
- Care to comment on perdurance of LET-23? It is not clear to me why the protein should be so rapidly cleared from the AC after initiation of FLP expression and deletion of the locus. Is LET-23 that labile? No perdurance in ventral uterine cells? The promoter must come on after the LIN-3 patterning of VPC fates, and so not that much time elapses before the described morphogenetic events.
- Also, this LET-23 tag does not perturb function? Gauthier and Rocheleau 2020 observed that a CRISPR tagged let-23 caused mild defects suggestive of weak loss of function. (Granted, not

© 2022. Published by The Company of Biologists under the terms of the Creative Commons Attribution License (https://creativecommons.org/licenses/by/4.0/).

necessarily problematic for this study were that true, though such could impact development prior to the developmental events being studied).

- I would prefer to see a second allele of the Netrin signal tested as a double with the conditional let-23 deletion, a standard validation of genetic experiments to avoid the pitfalls of single-allele genetics. Perhaps an allele of unc-40, which is the receptor of UNC-6, thus giving us a double receptor knockout?
- Much or all of section 1 belongs in methods and not results. The approach used here is a minor refinement of a previously published methods paper, applicable to the smaller temporal window of vulval development. Thus the methodology could be dramatically curtailed to include only refinements on the previous method, and relegated to the methods section and supplemental figures. Only the bare necessity required for reader comprehension should be left in the Results. As it stands this long section comes across as inflationary.

Minor details:

- Space between number and units (e.g. should be 1000 um and not 1000um.)
- Simpler to read if you split out your system validation from the actual observations. This is a big wall of text and a lot of explication that could be simplified by breaking it down into smaller bites in different sections.
- Worth noting for Discussion: other morphogenetic processes show delay rather than outright defect when one parallel arm of a control program is deleted. For example, cell corpse engulfment is often only delayed or partially penetrant defects with a single mutation, but completely penetrant with both branches knocked out. Also true for ingression of E.a and E.p gut blastomeres during gastrulation.
- Should “dots” be communicated as “punctae”?
- Misspellings: “assed,” “form” instead of “from”
- Line numbers would help the review process!

First revision

<u>Author response to reviewers' comments</u>

To reviewer 1:
Reviewer 1 Advance Summary and Potential Significance to Field:
During development of the C. elegans reproductive system, the uterine and vulva lumens must be connected through a process that involves the gonadal anchor cell (AC) invading through two basement membranes. Previous work has described AC invadopodia and various mechanisms that control its formation and placement. One gap in understanding has been the mechanism(s) that direct spatial placement of invadopodia so that the AC penetrates in the correct place. Here, Spiri et al provide an answer to this question.
Spiri et al. report for the first time expression of LET-23/EGFR in the gonadal AC and show a functional role for it there in aligning the AC to vulval cells for efficient basement membrane breaching and enlargement of the gap to join the vulva and uterus lumens. They show that a ring-shaped adherens junction forms between the AC and the vulval cells during alignment/breaching, and that LET-23 is required for its formation and for actin and junction dynamics associated with this appearance of this structure. Since LIN-3/EGF is expressed in the P6.p-derived vulva cells that are known to be important for breaching, this suggests a model whereby vulval LIN-3/EGF signals to AC LET-23/EGFR to control actin dynamics, invadopodia placement, and ring-shaped junction assembly.
Spiri et al also report on new, widely applicable, microfluidics device for short-term imaging of immobilized larvae. I predict this will be widely adopted. I can’t wait to contact the authors to request a prototype and detailed directions for making it! I also encourage the authors to find a vendor to commercialize it.

Regarding commercialization, we currently have no plans on making these devices a commercial product, but we may reevaluate this if sufficient demand is generated by the *C. elegans* community. For the time being we are happy to supply devices or master molds to anyone interested on a collaborative basis, with various other device sizes for different developmental stages available as well (mid L3 all the way to 2 day adulthood). Detailed instructions for master

© 2022. Published by The Company of Biologists under the terms of the Creative Commons Attribution License (https://creativecommons.org/licenses/by/4.0/).

mold and device fabrication, as well as device operation can already be found in the supplementary information (along with the required CAD files), and we are happy to give further information and assistance if needed.

C. elegans vulva development is a textbook example for how signaling controls different aspects of organ formation. The data in this manuscript will become part of this narrative that every review article, textbook entry, and class lecture on the topic will include. The results are clearly presented, the data appear robust.

Reviewer 1 Comments for the Author:
My only substantive comment is that the introduction could better set up the major gap in knowledge that the data will end up filling. Currently, the intro talks about too many other things besides the issue at hand, and it is not until the Discussion that authors mention the early results of Sherwood et al which showed the (unexplained) requirement for P6.p and the ability of P6.p-derived signals to act at a distance.

We have addressed this issue in the introduction and now point out the major gap in knowledge more clearly. (**lines 81-88**)

On my list of "experiments the reviewer would have loved to see but which are not essential for the authors to do": Look at dig-1 mutants or repeat VPC laser ablations of Sherwood et al. in the LET-23 AC-KO – prediction is that displacement or removal of P6.p-derived signals should not have any further impact.

Although this would be an interesting experiment, due to time constraints and given the additional experiments that were required (see reviewers 2 & 3) we could not conduct additional experiment.

To Reviewer 2:
Reviewer 2 Advance Summary and Potential Significance to Field:
In this manuscript Spiri et al examine the role of the EGFR, LET-23, in regulating C. elegans vulval morphogenesis. They describe and utilize an innovative microfluidics system for high-throughput imaging of L3 and L4 stage animals which should be useful for the community studying post- embryonic development. Notably, the authors identify a key-role for LIN-3/LET-23 reciprocal signaling post-fate specification as part of the previously unknown vulval cue. Specifically, the authors describe a novel role for LET-23 function autonomously in the AC, initially functioning in an epistatic relationship with Netrin/UNC-6/UNC-40 signaling as a guidance cue facilitating AC invasion, with FLP-mediated excision of endogenous LET-23 in the AC demonstrating a requirement for AC expression of LET-23 for several aspects of uterine-vulval morphogenesis, including proper AC alignment to the 1° fated VPCs, proper timing of BM breach, and proper organization of adherens junctions coordinated with the F-actin cytoskeleton in relation to the connection between the innermost vulF cells and the AC following invasion.

Reviewer 2 Comments for the Author:
In general, I am supportive of the publication of this manuscript but have several suggestions that might improve the manuscript as well as the incorporation of relevant literature that is missing from the current manuscript.
1. Perhaps the most important or relevant literature that needs to be incorporated throughout the manuscript is Naegeli et al. 2018 (doi.org/10.1016/j.devcel.2017.10.024). There are several important aspects of AC biology that were identified in this paper that are likely relevant to the current manuscript including:

a. Following BM breach the AC generates a large protrusion in the first 55 minutes following AC invasion, peaking at 30 minutes post-breach. Was this phase of AC invasion/morphogenesis captured during timelapse in this study and if so, is it affected by loss of LET-23 in the AC? It would be important/interesting to know if the multiple protrusions captured in Figure 3H in LET-23KO ACs are a result of a loss of the main protrusion of reflective of the AC continuing to generate invadopodia post-invasion rather than switching machinery to the

© 2022. Published by The Company of Biologists under the terms of the Creative Commons Attribution License (https://creativecommons.org/licenses/by/4.0/).

lysosome- dependent large protrusion likely utilized to facilitate initial BM gap expansion.

We thank the Reviewer for this important suggestion. Upon reevaluating our initial time-lapse experiment, in which this phase of AC invasion/morphogenesis was indeed captured, we found that the temporal resolution (15 minutes) as well as the image quality were not good enough to draw a firm conclusion. Thus, we conducted new time-lapse experiments with shorter acquisition intervals (5 minutes) and used a spinning disk system to obtain confocal image quality. We analyzed the volume of the main protrusion labelled with the *qyIs24[Pcdh-3>mCherry::PLCδ^{PH}]* reporter to label the actin-rich protrusions. Using a similar quantification as described in Naegeli et al., we found that the volume of the main protrusion in *let-23AcKO* animals was significantly smaller and expanded less rapid after basement membrane breaching. We have added these new data to the revised Fig.3 (**Fig.3F-H**) and supplementary Fig.S3 (**Fig.S3B**,**C**) and provide representative Movie files (**Movie 1**,**2**). The paragraphs describing this new experiment can be found on **lines 290-303** in the results and on **lines 681- 701** in the methods section.

b. *Endogenous Netrin/UNC-6 localizes to the 1° VPCs and is important for the orientation of the main protrusion generated by the AC in an UNC-40-dependent manner.*

We adapted this and added the corresponding references Naegeli et al. (2018) in the introduction (line 85), the result section (**lines 321-326**) and to Wang et al. (2014) in the discussion (**lines 500-503**).

c. *If an invasive protrusion is generated in LET-23KO animals, are characterized aspects of the invasive protrusion still localized to it in the absence of LET-23, including the exocyst complex (SNAP-29).*

To address this, we generated a reporter for SNAP-29 (*zhIs176[Plin-29>mKate::snap-29::unc-54 3`UTR II*]) *according to* Naegeli et al. (2018) and analyzed the expression pattern in *let-23AcKO* mutants. Similar to what we found observing the actin marker in the time-lapse experiments, *let-23AcKO* animals had smaller less focused invasive protrusions, but the overall distribution of SNAP-29 in the AC was not changed significantly. We therefore decided not to include these data in the revised manuscript. For illustration to this reviewer, representative images of two control and *let-23AcKO* animals (**A**) during the Pn.px-Pn.pxx as well as (**B**) two control and *let-23AcKO* animals during the Pn.pxx-L4.0 stages are shown here.

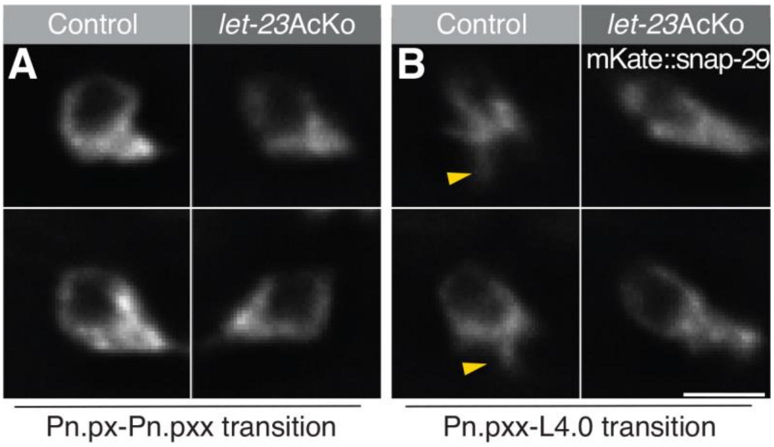

d. *In addition to the F-actin ring structure described in Figure 6 of this manuscript, there is an initial F-actin ring generated in the AC (see Fig. 6B of Naegeli et al 2018) during protrusion formation. Is this F-actin ring still generated in LET-23KO animals? Is the dystroglycan-mediated barrier that separates protrusion from apical-lateral AC plasma membrane intact of perturbed in LET-23KO animals?*

© 2022. Published by The Company of Biologists under the terms of the Creative Commons Attribution License (https://creativecommons.org/licenses/by/4.0/).

To address if the initial F-actin ring in the AC is still present in *let-23AcKO* mutants, *rol-6(d)* arrays were introduced to obtain images of dorso-ventrally oriented animals in the short-term microfluidic devices. Confocal images of ventrally oriented animals revealed that in addition to the L4.3, also in the L4.0 substage formation of the F-acting ring is less robust in *let-23AcKO* mutants, possibly influencing the alignment of the AC with vulF. We have updated Fig.6 (**Fig.6D-F**) as well as supplementary Fig.6 (**Fig.S6A**,**B)** with this new dataset. The text in the results section was adapted (**lines 417-433**) as well as the methods section (**lines 730-745**).

The DGN-1 (*qy18[dgn-1::mNG]*) expression pattern was likewise analyzed in *let-23AcKO* animals, but no obvious difference of the dystroglycan-mediated barrier between the protrusion and the apical lateral AC plasma membrane was found. Thus, this data has not been included in the revised manuscript.

2. Is it possible to trap animals oriented dorsal/ventral in the microfluidics device (which seems like it will be incredibly useful for the field!) – then rather than using xz reconstructions and losing most of the resolution in imaging, you would be able to image at high resolution in ventral- oriented animals. This would make visualization and quantification of the later steps of vulval morphogenesis much easier. If the microfluidics device is incapable of capturing animals in this orientation, one can use coverslips to roll animals to a ventral orientation to capture these dynamics at high-resolution on spinning-disc confocal or even inject rol-6 or utilize non-excised SEC-animals, which ~25% of the time will be ventral up on a silde.

Animals may to some extent be oriented dorsal/ventral using the device or much rather a modified version of it. In the device presented so far, most animals orient laterally due to carefully chosen channel aspect ratio. If this aspect ratio is inverted a larger percentage of animals will orient dorsal/ventral. It is possible to trap Roller animals in a ventral orientation in the current device to obtain higher resolution images of the actin ring, as described above under point **1.d**. The ventral-up ratio when loaded into the devices was comparable to animals on agar pads, but using the microfluidics short-term devices significantly reduced acquisition time.

Reviere 2 minor comments:
1. Page 3: avoid terms like “higher” and “lower” organisms – could use vertebrate vs invertebrates, but all of these organisms are extant and at the tips of their respective evolutionary lineage.

This has been changed in the introduction (**line 59**).

2. Figure 3: Consider using a red/green color-blind friendly heat map instead of the one used in Figure H’/G’.

This has been addressed, replacing the RGB with a suitable color palette in revised Fig. 3 (**Fig.3I`,J`**).

To Reviewer 3:
Reviewer 3 Advance Summary and Potential Significance to Field:
The manuscript entitled “Reciprocal EGFR signaling in the Anchor Cell ensures precise inter-organ connection during C. elegans vulval morphogenesis” (DEVELOP-2021-199900v1) describes the consequences of conditional deletion of the let-23 locus, which encodes the C. elegans EGFR-like protein, in the anchor cell (AC). This experiment led to the interesting observation of mis-positioning of the AC relative to the developing vulva and defects in the fusion of the two. This result is interesting because it represents a reciprocal signal of the developing primary lineage in the vulva back to the AC, which is the source of LIN-3/EGF that initially patterns the cell fates of the vulva, playing on theme of temporal iteration of the same signaling modules during development, the latter ones masked by the necessity of the earlier. Of additional interest is the finding that that the LET-23 signal in the AC proposed to govern invasion through the basement membrane is partially redundant with the Netrin signal originally described by the Sherwood lab. This work contributes to our understanding of developmental processes that refine precisely coordinated invasive cell movements during organogenesis, as well as insights into developmental

© 2022. Published by The Company of Biologists under the terms of the Creative Commons Attribution License (https://creativecommons.org/licenses/by/4.0/).

homeostasis and/or fidelity: how organisms account for positional variation or size differences to still generate proper forms.

Reviewer 3 Comments for the Author:
Reviewer 3 Major concerns

– *Regarding the main assay of the centering of the AC on vulval lineages: this is done by imaging of tagged HMR-1/beta-catenin to visualize adherens junctions of vulval lineages and an actin marker to visualize the AC. But how is the center point of each defined? We learn from the methods that this measurement is performed using a "semi-automated" process governed by a Fiji script. But we do not receive more information on this measurement, which underpins the most central assay of the paper. By visual inspection this assay is dubious: looking at the figures provided, it is unclear to this reviewer how the "center" of the AC or stack of toroids in the developing vulval could be defined. In some the placement of the "center of gravity" seems entirely subjective. Would these points in the developing animal be better defined by DIC for nuclei and the lumen of the invaginating pre-vulva? Or perhaps a combination of DIC and fluorescence?*

As indicated in the method section the center of the AC and toroids in the developing vulva is calculated from manually placed landmarks found in HMR-1::GFP images as part of a semi-automated analysis script. The user selects the landmarks shown as red, orange and magenta asterisks in **Fig. 3C**. Thereafter, the various indicated features, such as the mid-point of the AC (**blue asterisk in Fig. 3C**) are calculated automatically for each animal. These landmarks *are generally clearly recognizable as bright dots in the mid-sagittal sections of the HMR- 1::GFP fluorescence images, such that additional DIC images should not be necessary.*
Manual landmark placement (both, in fluorescence or DIC images) is of course associated with a certain error. However, as the used landmarks are on average 4.1 pixels wide, significantly smaller than the measured features (VulA1-VulA2: 254.3 pixels and AC diameter: 29.9 pixels), potential placement errors should have a minimal effect on the measured feature size and the made conclusions. By comparison, the average pixel size of the AC nucleus in the DIC images is 10.9 pixels. Hence, determining the mid-AC and also mid-vulF landmarks in DIC images as precisely as in the fluorescence images would be challenging. Measuring these landmarks in DIC images rather than the used fluorescence images is, in our opinion, more prone to possible placement errors.
The reviewer also remarked that "*In some the placement of the "center of gravity" seems entirely subjective*". We assume this refers to **Fig. 5A,** where the axis of each developing vulva is indicated by a dashed line. These indeed are unfortunately misplaced in some images, resulting in a discrepancy between the effective vulva center of gravity and alignment axis. The misplacement occurred during figure generation only and is not associated with any measurement error. Correct axes based on the actual measurements that are described above and were used to generate the plots shown in **Fig. 5C-F** are now indicated in the revised version of **Fig.5A** and **B,** and in the figure legend this point has been clarified (**lines 1098- 1099**).

– *Given the centrality of this assay to the conclusions of this study, it behooves the authors to better define how these numbers are determined. This assay lies at the core of this manuscript and the analysis that flows from the assay. If a user is arbitrarily defining those midpoints in an assay with relatively small difference between experimental and control conditions, it seems like a source of subjectivity in the data analysis.*

As stated above, measurement landmarks are generally clearly recognizable and significantly smaller than the measured features such that small variation in landmark placement should not result in measurement differences. The measurement method has been further clarified in the legend to **Fig. 3C** (**lines 1040-1044**), the Materials and Methods section (**lines 657-671**). The Fiji script used to obtain these measurement has been added to the supplementary information.

– *I looked for notation that these values were assigned in blind assays, but did not find them. But looking at Figure 3, the slightest bit of subjectivity could result in the entire difference*

© 2022. Published by The Company of Biologists under the terms of the Creative Commons Attribution License (https://creativecommons.org/licenses/by/4.0/).

we see. I would have much higher confidence if images had been coded and scored blindly.

Assays were not performed blind. As stated above, the used landmarks are clearly visible and significantly smaller than the features measured to calculate AC positioining. Hence, errors in manual landmark placement are very unlikely to result in a significant measurement error.

– *So while the methods section says this process is semi-automated using Fiji, either the quantification process needs to be better documented or the data re-scored by a blind scorer. Any manual scoring with knowledge of genotypes could fatally skew the data.*

The description of the measurement method was updated to describe our approach in detail (**lines 657-671**), and the Fiji script used has been added to the **Supplementary Information**, such that readers can clearly follow every step of the analysis method used.

– *Also, is there a negative control assay for the FLP expression? Maybe the let-23AcKO but without FLP expression? Ectopic promoters or proteins are frequently sufficient to alter cell behavior somewhat, though perhaps less likely because zhIs146 is a single copy transgene. So what we are seeing would not necessarily due to deletion of LET-23, but rather could be ectopic expression of PACEL-pes-10 (the promoter) disrupting the system. Perhaps the key control here would be the effects of zhIs146 on centering of vulva and AC in the absence of the let-23AcKO construct, thus showing that the effect depends on deletion of let-23 sequences. Or not!*

We agree with this reviewer that negative controls are essential. To test if the FLP transgene may have an effect, AC alignment in *zhIs146[pacel-pes-10>2xNLS-FLP-D5]* animals without the *let-23::FRT* allele (*zh131*) was analyzed. We updated **Fig. 3D,E** with new data including these negative controls and can rule out an effect of the *zhIs146* transgene alone. The result section was adapted accordingly (**lines 275-279**) as well as the legend to **Fig. 3 D,E** (**lines 1044- 1048**).

– *Care to comment on perdurance of LET-23? It is not clear to me why the protein should be so rapidly cleared from the AC after initiation of FLP expression and deletion of the locus. Is LET- 23 that labile? No perdurance in ventral uterine cells? The promoter must come on after the LIN-3 patterning of VPC fates, and so not that much time elapses before the described morphogenetic events.*

As shown in **Fig.S1A** and **B**, the *Acel::pes10::Flp* driver is already active in the AC and the VU early during AC fate specification in mid L2 larvae, before the onset of VPC fate patterning in late L2/early L3 (**Fig.S1C**). Since LET-23::GFP was only detected at later stages after induction during vulval morphogenesis, beginning in L4.0 (**lines 140-142**), the *let-23* gene has most *likely already been inactivated before it normally starts to be transcribed in the AC. Thus, no functional protein of LET-23 should have been present in the AC of* let-23AcKO *animals.*

– *Also, this LET-23 tag does not perturb function? Gauthier and Rocheleau 2020 observed that a CRISPR tagged let-23 caused mild defects suggestive of weak loss of function. (Granted, not necessarily problematic for this study were that true, though such could impact development prior to the developmental events being studied).*

This is a good point. To test if the *zh131* allele reduces LET-23 function, we have determined the vulval induction index of *zh131* animals and found a VI of 3.0 (n=44) exactly the same as in the wild-type controls (n=44). An important difference may be that Gauthier and Rocheleu 2020 used *mKate2* instead of GFP to tag *let-23*. In our experience, the type of fluorophore chosen to insert into a locus, even if inserted into the exact same location, can influence the function of the resulting fusion-protein. Inserting different fluorophores into the same location of *let-23* may lead to differences in protein function, thus creating weak *loss/gain of function* alleles. We have added the data on vulval induction in *zh131* into the result section and discuss this point briefly (**lines 128-137**).

© 2022. Published by The Company of Biologists under the terms of the Creative Commons Attribution License (https://creativecommons.org/licenses/by/4.0/).

- *I would prefer to see a second allele of the Netrin signal tested as a double with the conditional let-23 deletion, a standard validation of genetic experiments to avoid the pitfalls of single- allele genetics. Perhaps an allele of unc-40, which is the receptor of UNC-6, thus giving us a double receptor knockout?*

  To address this point, we have analyzed AC invasion in *let-23AcKO*; *unc-40(e271lf)* double mutants and found that they also show decreased BM breaching at the L4.0 substage, indicating a delay in AC invasion. These new data have been added to **Fig. 4**. However, the difference did not persist in the L4.4 substage, as it was the case in *let-23AcKO*; *unc-6(e400lf)* double mutants (**Fig. 4J,K**). One possible explanation for the difference between *unc-6(lf)* and *unc-40(lf)* mutants, described by Wang et al. (2014), could be that the AC in *unc-6(lf)* mutants is completely depolarized and does not form protrusions, while the AC remains polarized in *unc40 (lf)* mutants and retains an undirected protrusive activity (Wang et al, 2014). The result section has been adapted accordingly (**lines 349-354**) as well as the figure legend (lines 1086- 1088). In the discussion, the difference between *unc-6(lf)* and *unc-40(lf)* is further discussed (**lines 497-503**).

- Much or all of section 1 belongs in methods and not results. The approach used here is a minor refinement of a previously published methods paper, applicable to the smaller temporal window of vulval development. Thus the methodology could be dramatically curtailed to include only refinements on the previous method, and relegated to the methods section and supplemental figures. Only the bare necessity required for reader comprehension should be left in the Results. As it stands this long section comes across as inflationary.

  We believe this paragraph is essential in the Results section, as the manuscript not only aims to display novel biology but the novel method through which it was observed. As Reviewers 1 and 2 have pointed out, we believe the short-term imaging method to become widely adapted by the *C. elegans* research community. A clear introduction of the method, the design rationale as well as the relevant literature background is therefore essential. While the short-term method is based on our long-term imaging method, short- and long-term imaging devices are not the same and follow different design parameters facilitating their respective function. If a reader were to try and fabricate short-term devices using the parameters given in the long- term imaging paper (or vice versa), the devices would be significantly harder to operate, if not impossible to use. As we want to facilitate adaptation of both methods, we think it is essential to make these differences as clear as possible, to avoid confusion and allow adaptation of each method to specific experimental requirements.

Reviewer 3 minor details:

- *Space between number and units (e.g. should be 1000 um and not 1000um).*

  This has been addressed.

- *Simpler to read if you split out your system validation from the actual observations. This is a big wall of text and a lot of explication that could be simplified by breaking it down into smaller bites in different sections.*

Please see above.

- *Worth noting for Discussion: other morphogenetic processes show delay rather than outright defect when one parallel arm of a control program is deleted. For example, cell corpse engulfment is often only delayed or partially penetrant defects with a single mutation, but completely penetrant with both branches knocked out. Also true for ingression of E.a and E.p gut blastomeres during gastrulation.*

We agree that this would be additional interesting points worth to discuss, unfortunately due to limitations in characters we were not able to insert a complete discussion of this issue. To leave enough room for the discussion of other crucial points we did not include this interesting

© 2022. Published by The Company of Biologists under the terms of the Creative Commons Attribution License (https://creativecommons.org/licenses/by/4.0/).

viewpoint into our final manuscript.

– *Should "dots" be communicated as "punctae"?*

This has been addressed (**lines 389, 403, 663 and 1044**).

– *Misspellings: "assed," "form" instead of "from"*

These typos have been addressed.

– *Line numbers would help the review process!*

Line numbers were added.

Second decision letter

MS ID#: DEVELOP/2021/199900

MS TITLE: Reciprocal EGFR signaling in the Anchor Cell ensures precise inter-organ connection during *C. elegans* vulval morphogenesis

AUTHORS: Silvan Spiri, Simon Berger, Louisa Mereu, Andrew deMello, and Alex Hajnal
ARTICLE TYPE: Research Article

I am happy to tell you that your manuscript has been accepted for publication in Development, pending our standard ethics checks.

Reviewer 2

Advance summary and potential significance to field

In this improved, revised manuscript the authors illustrate the role that EGF/EGFR signaling plays during uterine-vulval morphogenesis and anchor cell invasion. Their data fills in a gap in our understanding of how the AC responds to micro-environmental cues beyond the now well-characterized role of netrin signaling in guiding AC invasion. Additionally, they provide a new microfluidics device for use in live-cell imaging of post-embryonic stage *C. elegans*, a tool that should be useful to many in the *C. elegans* community.

Comments for the author

The authors have done an excellent job incorporating my and the other peer reviewers' suggestions and I think the manuscript is stronger for it!

Reviewer 3

Advance summary and potential significance to field

This resubmission of DEVELOP-2021-199900, now v2, shows responsive additions in data and writing to concerns raised by myself and other reviewers. The biological story of a retrograde EGFR signal controlling AC invasion, paralleling the established Netrin system, is of broad interesting in the developmental biology community. Additionally, the engineering of spatiotemporal deletion of endogenous LET-23/EGFR in the AC is a cutting edge approach to bypass the requirement of LET-23/EGFR in inducing the primary cell earlier in development, and thus represents an elegant means of specifically interrogating gene functions during development and bypassing previous barriers to such analysis.

© 2022. Published by The Company of Biologists under the terms of the Creative Commons Attribution License (https://creativecommons.org/licenses/by/4.0/).

Comments for the author

Very minor details for publication:

- Observe proper nomenclature for CRISPR alleles in *C. elegans* as designated by Dickinson, e.g. let-23(zh131[FRT::let-23::FRT::gfp]) rather than FRT::let-23::FRT::gfp(zh131)
- Reminder of genotype nomenclature, e.g. unc-40(e271lf), “lf” should not be italicized while the rest is italicized.

© 2022. Published by The Company of Biologists under the terms of the Creative Commons Attribution License (https://creativecommons.org/licenses/by/4.0/).
